# A Ratiometric Calcium Reporter CGf Reveals Calcium Dynamics Both in the Single Cell and Whole Plant Levels Under Heat Stress

**DOI:** 10.3389/fpls.2021.777975

**Published:** 2021-12-17

**Authors:** Chrystle Weigand, Su-Hwa Kim, Elizabeth Brown, Emily Medina, Moises Mares, Gad Miller, Jeffrey F. Harper, Won-Gyu Choi

**Affiliations:** ^1^Department of Biochemistry and Molecular Biology, University of Nevada, Reno, Reno, NV, United States; ^2^The Mina and Everard Goodman Faculty of Life Sciences, Bar Ilan University, Ramat-Gan, Israel

**Keywords:** calcium, mCherry fused GCaMP6f, whole rosette imaging, pollen tube imaging, single cell imaging, ratiometric calcium reporter CGf, heat stress

## Abstract

Land plants evolved to quickly sense and adapt to temperature changes, such as hot days and cold nights. Given that calcium (Ca^2+^) signaling networks are implicated in most abiotic stress responses, heat-triggered changes in cytosolic Ca^2+^ were investigated in *Arabidopsis* leaves and pollen. Plants were engineered with a reporter called CGf, a ratiometric, genetically encoded Ca^2+^ reporter with an m**C**herry reference domain fused to an intensiometric Ca^2+^ reporter **G**CaMP6**f**. Relative changes in [Ca^2+^]_cyt_ were estimated based on CGf’s apparent *K*_D_ around 220 nM. The ratiometric output provided an opportunity to compare Ca^2+^ dynamics between different tissues, cell types, or subcellular locations. In leaves, CGf detected heat-triggered cytosolic Ca^2+^ signals, comprised of three different signatures showing similarly rapid rates of Ca^2+^ influx followed by differing rates of efflux (50% durations ranging from 5 to 19 min). These heat-triggered Ca^2+^ signals were approximately 1.5-fold greater in magnitude than blue light-triggered signals in the same leaves. In contrast, growing pollen tubes showed two different heat-triggered responses. Exposure to heat caused tip-focused steady growth [Ca^2+^]_cyt_ oscillations to shift to a pattern characteristic of a growth arrest (22%), or an almost undetectable [Ca^2+^]_cyt_ (78%). Together, these contrasting examples of heat-triggered Ca^2+^ responses in leaves and pollen highlight the diversity of Ca^2+^ signals in plants, inviting speculations about their differing kinetic features and biological functions.

## One-Sentence Summary

This paper shows that heat stress can trigger cytosolic Ca^2+^ signals in seedling leaves and suppress the growth associated patterns of Ca^2+^ oscillations in pollen tubes.

## Introduction

An important adaptive trait for many land plants is an amazing ability to sense and adapt to changing temperatures (Lamers et al., 2020; Hayes et al., 2021; Mareri et al., 2021; Nishad and Nandi, 2021). Nevertheless, global climate change is predicted to make periods of heat stress increasingly detrimental to plant growth and reproduction (Challinor et al., 2014; Zhao et al., 2017; Cohen et al., 2021; Zandalinas et al., 2021). The threshold at which different plants succumb to heat stress varies and can be influenced by combinatorial stresses, such as drought, light intensity, and nutrition (Lamers et al., 2020; Hayes et al., 2021; Zandalinas et al., 2021). In *Arabidopsis*, optimal growth occurs around 16–25°C (Calhoun et al., 2021). As temperatures rise to 30–37°C, *Arabidopsis* plants activate heat stress response pathways (Hayes et al., 2021). While long term exposure to temperatures around 40°C will ultimately cause cell death, *Arabidopsis* can still complete its life cycle with a diurnal stress regime that includes a 1-h mid-day 40°C heat stress, albeit with a major reduction in seed set (Tunc-Ozdemir et al., 2013).

As plants are exposed to heat stress, multiple cellular processes are disrupted, including protein folding, cytoskeletal organization, membrane stability, regulation of reactive oxygen species (ROS), and ion homeostasis (Lenzoni and Knight, 2019; Lamers et al., 2020; Zandalinas et al., 2020; Hayes et al., 2021). Multiple mechanisms for heat sensing have been proposed, including direct changes to membrane fluidity, photosensors, and transcription factors (Lamers et al., 2020; Zandalinas et al., 2020; Hayes et al., 2021). Temperature sensing likely occurs independently in different organelles, for example, in the chloroplast and ER (Lenzoni and Knight, 2019; Malini et al., 2020; Li and Howell, 2021; Singh et al., 2021). In theory, most macromolecules in a cell can be structurally or kinetically altered by heat, which invites consideration that heat sensing thresholds might occur as meta-phenomena that evolved without dedicated sensors.

Ca^2+^ signaling networks are implicated in most abiotic stress responses in plants (Atif et al., 2019; Tang et al., 2020; Alves et al., 2021; Ma et al., 2021; Noman et al., 2021). However, there is mixed evidence to directly support a role for Ca^2+^ signals as an initial heat sensing response (Gao et al., 2012; Tunc-Ozdemir et al., 2013; Finka and Goloubinoff, 2014; Lenzoni and Knight, 2019). For example, a recent study failed to detect a heat-triggered Ca^2+^ signal in the cytosol of cotyledon staged seedlings, but did observe a strong signal in the chloroplast (Lenzoni and Knight, 2019). Some of the mixed results might be explained by a reliance on the Ca^2+^ reporter aequorin. Aequorin has relatively weak affinity for Ca^2+^ (*K*_D_ ∼ 7–13 μM) (Costa et al., 2018), which makes it suboptimal for detecting Ca^2+^ signals in the low to mid nM range (i.e., near resting [Ca^2+^]_cyt_ around 50–100 nM).

Here, we used a new design for a genetically encoded ratiometric Ca^2+^ reporter to investigate heat-triggered [Ca^2+^]_cyt_ changes in leaves and pollen tubes from *Arabidopsis thaliana.* This reporter, called CGf, was engineered with an m**C**herry fused to the N-terminal end of an intensiometric Ca^2+^ reporter **G**CaMP6**f**, and incorporates a ratio design feature similar to other ratiometric reporters comprised of two tandem fluorescent proteins (Cho et al., 2017; Waadt et al., 2017; Luo et al., 2019). The Ca^2+^ sensor domain is derived from GCaMP6f, which is a well-established Ca^2+^ reporter with a *K*_D_ = 220–375 nM (Chen et al., 2013; Badura et al., 2014; Helassa et al., 2016; Costa et al., 2018).

While GCaMP Ca^2+^ reporters are useful for detecting qualitative changes in [Ca^2+^]_cyt_, without an internal reference for normalization, it is sometimes difficult to know whether intensity differences might be caused by varying levels of reporter expression or localization rather than changes in Ca^2+^ dynamics. Using mCherry as an internal reference, CGf’s ratiometric feature provides an opportunity to compare Ca^2+^ signaling between different tissues, cell types, or subcellular locations. CGf was used here to reveal three different heat-triggered cytosolic Ca^2+^ signals in leaves, as well as a very different heat-induced suppression of tip-focused Ca^2+^ oscillations in growing pollen tubes. The observation that pollen failed to show the same heat stress signals as seen in leaves highlights the need to consider how different plant cells sense and respond to heat.

## Materials and Methods

### Plant Materials and Growth Conditions

Hygromycin-resistant *Ubiquitin10promoter:mCherry-GCaMP6f* (plasmid stock 2935) was transformed into *Arabidopsis thaliana* COL-0 using *Agrobacterium tumefaciens* (GV3101 strain) floral dip method (Clough and Bent, 1998). Sterilized seeds were sown on square Petri dishes containing 0.5x Murashige and Skoog medium (Phytotechnology Laboratories, pH 5.7), 0.05% (w/v) MES, 25 mg/L hygromycin B (Gold Biotechnology), and 1% (w/v) agar. After 48–72 h of stratification in the dark at 4°C, seeds were transferred to room temperature conditions with constant light for 10 days. Thereafter, seedlings were transplanted into soil prepared according to manufacturer guidelines (Sunshine SMB-238 SunGro Horticulture, Marathon pesticide, Cleary Turf and Ornamental Systemic Fungicide). Plants were grown to maturity in growth chambers at 22°C with 70% humidity and 16 h light (∼125 μmol m^–2^ s^–1^) followed by 8 h dark. For whole-plant heat stress imaging, seedlings grown in the above conditions for 10 days were transplanted to hydrated 36 mm diameter Jiffy-7 Peat pellets (Jiffy group, Manitoba, Canada) and placed in Turface MVP (Profile Products LLC, Buffalo Grove, IL) in Magenta vessel GA-7-3 (MilliporeSigma, Burlington, MA). Plants grew additional 2 weeks (or until ready for imaging) under long day light cycles (16 h-light/8 h-dark) at 22°C.

### Plasmid Construction

For plant expression, *Ubiquitin10promoter:mCherry-GCaMP6f* (plasmid stock 2935) was constructed through standard molecular techniques using a pGreenII vector system (Hellens et al., 2000) with a hygromycin resistant (HygR) selection marker for plants and a kanamycin resistance (KanR) selection marker for *E. coli*. For *in vitro* analyses, coding sequence for *mCherry-GCaMP6f* (plasmid stock 3007) and *GCaMP6f* (plasmid stock 3221) were cloned separately into a KanR pET28-Novagen vector (Sigma-Aldrich). Promoters used included a Ubiquitin 10 promoter (UBQ10; Norris et al., 1993) for pGreenII vector and a T7 lac promoter (Studier and Moffatt, 1986) for pET28 vectors. The DNA sequence is provided in Supplementary Figure 5 for *mCherry-GCaMP6f* (plasmid stock 2935).

### Genetics and Seed Set Analyses

Transgene transmission was measured by scoring hygromycin resistance of F1 progeny from reciprocal outcrosses. Seeds were processed as described in section “Plant materials and Growth Conditions.” Statistical significance was determined using Pearson’s chi-squared test (*X*^2^) unless stated otherwise. For seed set analyses, mature siliques were cleared by incubation in 70% ethanol at room temperature over 24 h.

### Imaging Equipment

Whole-plant images were collected using an AxioZoom V16 fluorescent microscope with a PlanNeoFluar Z 1.0x/0.25x objective (Carl Zeiss, Inc., Thornwood, NY, United States) and ORCA-Flash4.0 V2 Plus sCMOS digital camera (Hamamatsu Photonics Inc., San Jose, CA). For ratio imaging, separate signals from GCaMP6f and mCherry domains were detected with filter set 38 eGFP shift free (Ex 470/40 nm, dichroic mirror 495 nm; Em 525/50 nm) and filter set 63 HE mRFP shift free (Ex 572/25 nm, dichroic mirror 590 nm; Em 629/62 nm).

The same imaging equipment was used for pollen heat stress assays except with a PlanNeoFluar Z 2.3x/0.57x objective and a W-view Gemini Image Splitting Optics for simultaneous two fluorescence imaging (Hamamatsu Photonics Inc., San Jose, CA) equipped with emission filter sets (Chroma ET510/20 nm 25 mm diameter for GFP signal and Chroma ET632/60 nm 25 mm diameter for mCherry signal) and a 25.5 × 36 × 2 mm (W x L x H) T560lpxr-UF2 dichroic mirror (Chroma, Bellows Falls, VT).

For high resolution imaging, pollen time lapse images were captured using a Leica DMi8 inverted microscope fitted with a Yokogawa CSU-W1 spinning disk confocal scanner module and a CCD camera. Images were captured with 63X/1.4 NA objective with filter switching (GFP laser Ex 488 nm Em 525 nm/50 m OD8; RFP laser Ex 561 nm Em 610 nm/75 m OD8).

### Rosette Imaging and Analysis of Blue Light- and Heat-Triggered Ca^2+^_cyt_ Increases

Blue light- and heat-triggered [Ca^2+^]_cyt_ changes were monitored using wild type COL-0 plants stably expressing CGf. Analyses shown were conducted using ∼3-week-old rosettes when plants showed a minimum of 8–9 true leaves. Plants were allowed to adapt to dim light for at least one hour prior to the start of a blue light exposure at time –120 min (Ex470/40nm with 24 μmol m-2s-1 intensity). Images corresponding to GCaMP6f and mCherry were obtained every 5 sec for 5 h.

Heat stress exposures were achieved by placing ∼3 week-old, soil-grown plants in a custom-made heat chamber with temperatures increasing to ∼40°C in ∼6 min after turning the heat pad on. The custom-made heat chamber was built by installing an 8 inch diameter digital heat pad at the bottom of a 4-inch high domed container constructed with a viewing port covered with thin plastic wrap (Supplementary Figure 4).

Fluorescent intensities of GCaMP6f and mCherry were acquired from individual GFP and RFP channel images at each time point using ImageJ software (Abràmoff et al., 2004). Intensity changes for GCaMP6f and mCherry were individually calculated using single wavelength quantification equations. For mCherry, the calculation was F_t_/F_basal_, where ‘F_t_’ is fluorescence measured at a given time point within the time course. For GCaMP6f, the calculation was ΔF/F_basal_, where ΔF is F_t_ -F_basal_, and F_basal_ is mean value of pre-heat period between –10 to 0 min. A CGf ratio was calculated as GCaMP6f fluorescent intensity (greenF_t_) divided by mCherry fluorescent intensity (redF_t_). Percentile (%) CGf max in leaves was calculated using a CGf max ratio divided by the CGf max ratio 2.15 (Supplementary Figure 3 and Supplementary Movie 2).

### *In vitro* Pollen Germination

*Arabidopsis* pollen grains from 1 to 2 day old flowers were germinated on an agar surface containing 1.5% low melting agarose, 10% (w/v) sucrose, 2 mM calcium chloride (CaCl_2_), 0.004% (w/v) boric acid (H_3_BO_3_), 2 mM potassium chloride (KCl), 0.4 mM magnesium sulfate (MgSO_4_), and pH 7.5 using potassium hydroxide (KOH). Pollen grains germinated in a humidity chamber in the dark at 22°C for 1–3 h. Glass coverslips were placed over pollen tubes on the agar surface prior to imaging. Only pollen tubes between 100 and 400 μm in length were used for analysis.

### Pollen Heat Stress Assay and Time Series Analysis

Pollen heat stress experiments were imaged using PlanNeoFluar Z described above in section “Imaging Equipment” Both red and green fluorescence signals were simultaneously imaged using an optical splitter at 500 ms frame^–1^ for 4 min. Control (no heat treatment) pollen tubes were imaged at room temperature (22°C). Time course for heat treatment was applied as follows: initial 22°C for 1 min, temperature ramping from 22 to 32°C in 1 min, and 32°C for remaining 2 min. Heat treatment was applied using a COSORI digitally controlled cup warmer (Catalog#CO194-CW).

Pollen time lapse images were adjusted using ImageJ plugins Rolling Ball Background Subtraction and 3D Drift Correction (Parslow et al., 2014). After image adjustment, Multiple Kymograph plugin was used to generate kymographs (average pixel neighborhood = 5) for individual fluorescent channels. Kymograph text files were analyzed using CHUKNORRIS web interface^1^ for single channel kymographs (Damineli et al., 2017). A raw ratio was calculated using CHUKNORRIS-derived time series (ROI.ts) data by dividing GCaMP6f fluorescent intensities (greenF_t_) by mCherry fluorescence (redF_t_). Raw ratios were converted to a % maximum of the CGf reporter using the maximum raw ratio observed during pollen tube bursting events.

High resolution pollen imaging was performed using a spinning disk confocal described above in section “Imaging Equipment” using 1 s intervals. Time series analysis was performed as described above. The region of interest for tip-focused imaging was 10 μm (pollen apex), whereas flicker ratios were determined from a 50 μm region behind the pollen apex.

### *E. coli* Protein Expression, Purification, and Characterization

CGf and single fluorescent GCaMP6f proteins were cloned into pET expression vector and transformed into T7 expression cells (NEB cat# C2566). Mid-log phase liquid cultures in 2xYT media were induced with 0.5 mM IPTG and grown at 30°C for 3 h. Cells were harvested by centrifugation and resuspended in lysis buffer (20 mM MOPS pH 7.2, 500 mM NaCl, 10% glycerol (w/v), 10 mM Imidazole, 1.5 mg/mL Lysozyme, 1 mM PMSF) and frozen at –20°C. Upon thawing, cells were lysed by the addition of 0.4% Triton x100 and sonication. Lysates were cleared by centrifugation at 10,000 rpm for 30 min at 4°C. Cleared cell lysate was applied to Ni-NTA beads (Qiagen, 700 μl packed bead volume) and were washed by 10 column volumes of each of the following buffers: 6xHis MOPS Wash Buffer (20 mM MOPS pH 7.2, 100 mM NaCl, 10 mM Imidazole), 6xHis MOPS Wash Buffer with 1 mM EGTA, and 6xHis MOPS Wash Buffer with 0 mM EGTA. Proteins were eluted with 200 mM Imidazole and concentrated using Pierce Concentrator 10K MWCO filters (Thermo Scientific). Eluted proteins were stored in 60% glycerol (w/v) and stored at –20°C.

Protein concentrations were measured using Bradford reagent (BioRad) and Pre-Diluted BSA Protein assay standards (Thermo Fisher Scientific) in 96 well plates analyzed by Spectromax M5 plate reader at 595 nm absorbance. Protein purity was determined using SDS-PAGE (AnyKD Bio-Rad) and total protein stain (Gel Code Blue, Thermo Fisher Scientific).

To measure *in vitro* Ca^2+^ binding affinities, fluorescence intensities were measured at various free [Ca^2+^] concentrations using a Calcium Calibration Buffer Kit (Invitrogen) with 30 mM MOPS pH 7.2, 100 mM KCL, and varying additions of 10 mM EGTA or 10 mM CaEGTA. Dilution series were performed as directed by the manufacturer’s protocol with absorbance measured using Shimatzu RF-6000 Spectrofluorometer. Fluorescence intensities were measured for GFP at Ex 488 nm/Em 512 nm.

### *In planta* Protein Stability Analysis

Leaf tissue from 10-day-old seedlings, grown as described in section “Plant Materials and Growth Conditions” was collected and frozen in liquid nitrogen. Frozen tissue was ground into a frozen powder using prechilled mortar and pestle, adding homogenization buffer (HB) as needed (i.e., 100 μL HB per 100 mg tissue). Homogenization buffer consists of 100 mM Tris pH 7.5, 150 mM NaCl, 290 mM sucrose, 10 mM imidazole, 10% glycerol, 0.1% Tween-20, EDTA-free protease inhibitor cocktail V (cat# P50900-1, Research Products International), and 1 mM PMSF (phenylmethylsulfonyl fluoride). Plant extracts were filtered through cheesecloth prewet with HB. Filtered extract was spun in glass Corex tubes at 6,000 g for 15 min at 4°C to remove cellular debris. Crude plant extracts were analyzed using SDS-PAGE precast gels (AnyKD, Bio-Rad), followed by Western blot analysis of reporters using primary RFP monoclonal antibody (Thermo Fisher cat # 200-301-379S) and secondary F(ab’)2-Goat anti-Mouse IgG (H + L) antibody (HOURP, Thermo Fisher Cat# A24512).

## Results

### A Fusion of mCherry to GCaMP6f Generates a Robust Ratiometric Ca^2+^ Reporter

**FIGURE 1:**
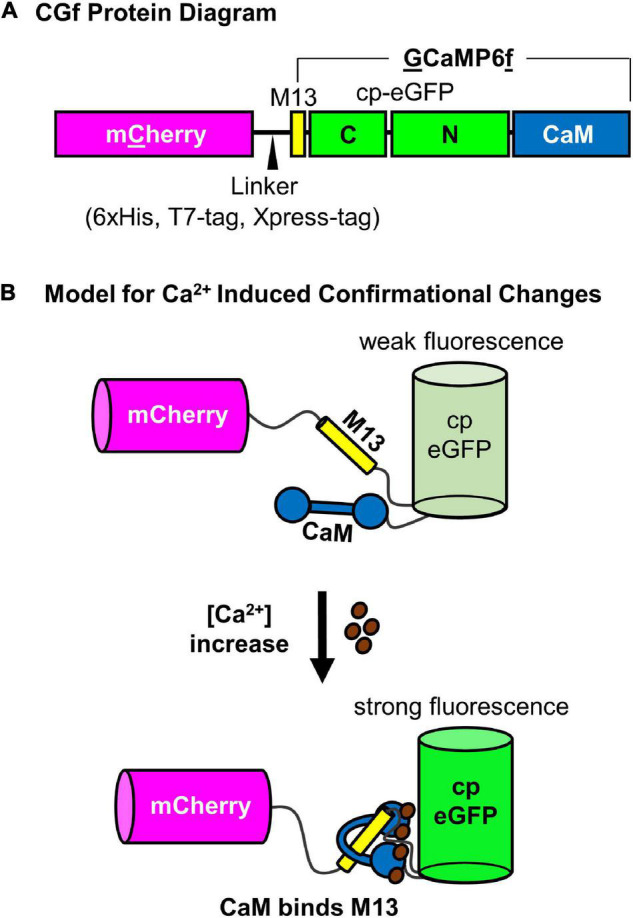
Schematic diagram of CGf, a ratiometric GCaMP6-based Ca^2+^ reporter. **(A)** CGf protein diagram of m**C**herry fusion to N-terminal end of **G**CaMP6**f**, which is comprised of an M13 binding site for calmodulin, a cp-eGFP, and calmodulin (CaM). The linker between mCherry and GCaMP6f includes a 6xHis, T7-Tag, and Xpress Tag (MedChemExpress, LLC). **(B)** Model for Ca^2+^ induced conformational changes. (Top) Without Ca^2+^, the calmodulin domain is dissociated from the M13 peptide, which results in a conformation that allows solvent access to protonate the chromophore and quench eGFP fluorescence (weak fluorescence) (Ding et al., 2014). As [Ca^2+^] increases, Ca^2+^ binding to CaM promotes CaM-M13 binding interactions, which blocks solvent access, promotes deprotonation of the chromophore, and increases eGFP fluorescence (strong fluorescence). mCherry fluorescence is not dependent on Ca^2+^ binding, serving as an internal reference for comparison to the Ca^2+^ dependent changes in eGFP fluorescence.

**FIGURE 2:**
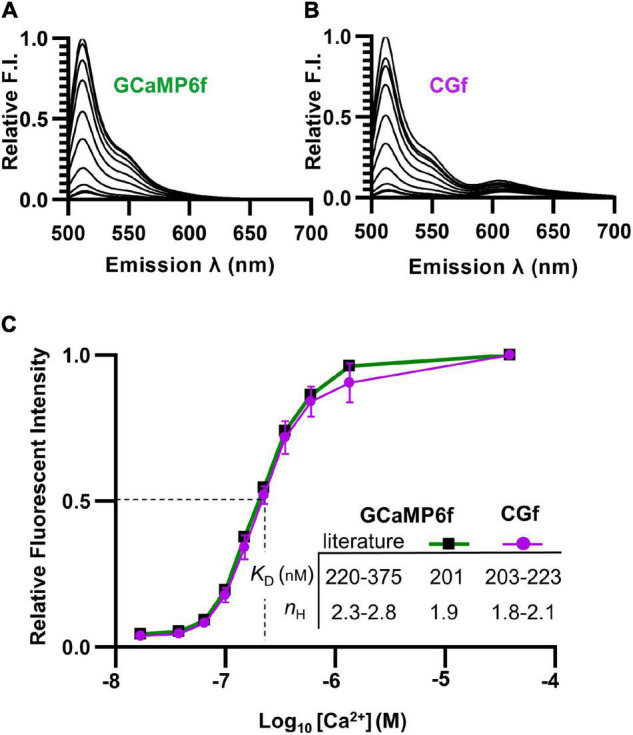
Spectral comparisons showing that the fusion of mCherry to GCaMP6f did not alter Ca^2+^ binding. **(A,B)** Emission spectra comparing relative brightness of GCaMP6f **(A)** to CGf **(B)** across varying Ca^2+^ concentrations from 0.017 to 39 μM. **(C)** Ca^2+^ titration curves comparing GCaMP6f to CGf. These results were used to calculate Ca^2+^ binding affinities (apparent *K*_D_) and Hill equation fits (*n*_*H*_). Comparisons are provided to previously reported ranges for GCaMP6f (Chen et al., 2013; Badura et al., 2014; Helassa et al., 2016).

A ratiometric Ca^2+^ reporter was generated with an N-terminal m**C**herry followed by a linker and an intensiometric Ca^2+^ reporter **G**CaMP6**f** (which has a calmodulin binding sequence “M13” followed by a circularly permutated eGFP and a terminal calmodulin domain (Chen et al., 2013; Figure 1A). When Ca^2+^ binds to the calmodulin domain, the protein undergoes a conformational change that promotes intra-molecular binding to the M13 peptide sequence (Figure 1B). This conformational change results in an increase in GFP fluorescence proportional to [Ca^2+^] (Figure 2).

To determine whether the Ca^2+^ affinity of GCaMP6f was altered by a fusion with mCherry, both CGf and an unfused GCaMP6f reporter were separately expressed in *E. coli* and purified. Spectral analyses indicated that both CGf and GCaMP6f showed very similar Ca^2+^ titration curves (Figures 2A,B). CGf bound Ca^2+^ with an apparent *K*_D_ in the range of 203–223 nM (Figure 2C), which is similar to other measurements in the literature for GCaMP6f that ranged from 220 to 375 nM (Chen et al., 2013; Badura et al., 2014; Helassa et al., 2016; Costa et al., 2018). An approximate *K*_D_ of 220 nM is used here as a point of overlap between our measured *K*_D_ and the lower end of the range reported in the literature. The Hill coefficients for both CGf and GCaMP6f were in the range of 1.8–2.1 (Figure 2C), which indicates that the designed fusion did not significantly alter the expected cooperative binding of Ca^2+^ to the calmodulin domain. These Ca^2+^ binding analyses indicate that the addition of an mCherry to the N-terminal end of GCaMP6f did not dramatically alter basic kinetic features previously established for GCaMP6f.

To address the potential concern that the mCherry domain might dampen GCaMP fluorescence because of Förster resonance energy transfer (FRET), the spectra for the Ca^2+^ titration curves was evaluated over an expanded emission range to include potential fluorescence from mCherry (emission 600–650 nm) (Figures 2A,B). An alternative fusion design was previously reported to show energy transfer to mCherry and up to a 50% quenching of the GCaMP6 signal (Cho et al., 2017; Luo et al., 2019). In contrast, the CGf design here showed relatively little evidence of quenching, with an estimated 1.3% of the total spectral fluorescence (at the *K*_D_ [Ca^2+^] of 220 nM) resulting from a potential Ca^2+^ dependent energy transfer from GCaMP6f to mCherry (Figure 2B, emissions between 600 and 650 nm). Thus, in the context of using the mCherry fluorescence as a normalization baseline, this small amount of FRET (1.3%) did not appear to represent a major concern.

To evaluate whether the CGf fusion was proteolytically stable when expressed in plants, stable transgenic plants were generated that expressed CGf under the control of a UBQ10 promoter. Plants were chosen for analysis using fluorescence microscopy to confirm strong expression throughout the plant, including leaves and pollen. Protein extracts from leaves were subjected to SDS-PAGE. A Western blot analysis was conducted using a primary antibody recognizing mCherry. A single band was detected at a size expected for an intact mCherry-GCaMP6f fusion (Supplementary Figure 1). This provided corroboration that the fusion was proteolytically stable, which was considered important because a confident interpretation of the ratiometric output for CGf relies on the maintenance of a 1:1 stoichiometry between the reference mCherry and CGaMP6f.

### CGf Can Be Expressed Without Disrupting Plant Development

A general concern for constitutive expression of any bioreporter is whether the reporter itself might significantly impact an organism’s development or responses to environmental stimuli. This concern was especially relevant here because previous reports of potential phenotypic impacts were reported from the over-expression of calmodulin (Yang et al., 2018), as well as several Ca^2+^ reporters that harbor a calmodulin domain (Costa et al., 2018). However, for the CGf construct used here, the frequency of plants with obvious phenotypes appeared to be less than 1 in 10.

**FIGURE 3:**
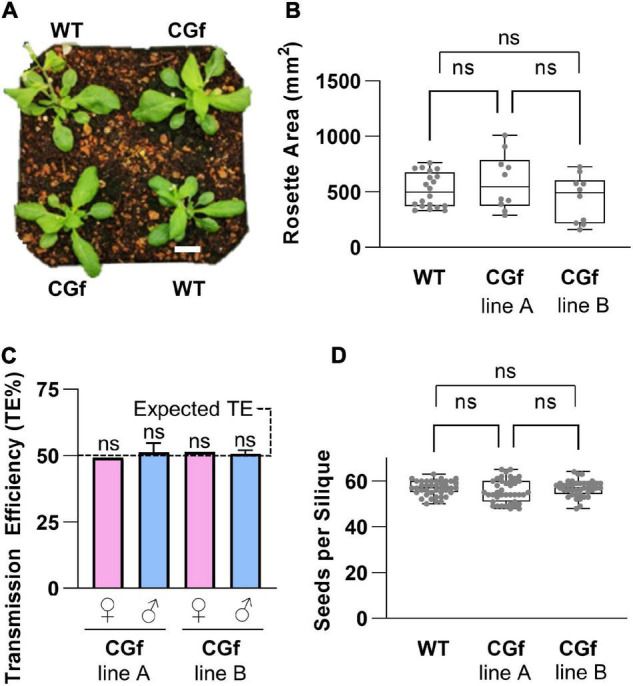
CGf expression did not alter plant development and reproduction. **(A)** Visual representation of 5-week-old wild type (WT) and CGf plants at the time of bolting. Scale bar = 5 mm. **(B)** Total rosette area of 5-week-old plants. WT *n* = 20; Two independent CGf lines **(A,B)** *n* = 10 each; from 3 experimental replicates. **(C)** CGf genetics. Reciprocal outcrosses were performed on plants harboring a heterozygous hygromycin-resistant transgene. Progeny were scored for hygromycin resistance to determine transmission efficiency (TE) of the transgene. TE = # resistant seedlings/total # seedlings. Female (♀) or male (♂) refers to outcross transmission of gamete harboring CGf transgene. WT pollen were outcrossed to CGf ovules. CGf pollen were outcrossed to *male sterility 1* (*ms1-1*) plants. TE% was not significantly (ns) different than expected 50% TE, as determined by the Pearson’s Chi-square test (χ^2^) with a minimum threshold set at *p* ≤ 0.05. ♀ *n* > 50; ♂ *n* > 300 for each independent line (seed stocks 2540 and 2640). **(D)** Seed set analyses showing no statistical significance between WT and CGf. *n* = 40 siliques for each group; 3 replicates. NS determined using One-way ANOVA multiple comparisons with the Turkey post-hock test. Error bars show min to max values of representing data sets (gray dots). Unless stated otherwise, CGf line A is seed stock (ss) 2543. CGf line B is ss2544.

For the selection of plant lines for Ca^2+^ imaging, additional characterizations were done to confirm the absence of serious phenotypic problems (Figure 3). With the same transgenic lines used for imaging rosettes, plants were grown side by side in the same pots with wild type controls (Figure 3A). In these paired growth comparisons, we failed to observe any statistical differences in rosette sizes, root growth on agar plates, average seed numbers per silique, or total seed yield per plant (Figure 3 and Supplementary Figure 2). Similarly, for transgenic lines used to image pollen, pollen outcrosses from heterozygous plants failed to reveal any non-Mendelian distortion in pollen transmission efficiencies (Figure 3C).

As commonly observed for many transgenic plants, some of the plant lines maintained over multiple generations appeared to segregate rare examples of gene silencing, as detected by screening seedlings for consistently high levels of mCherry fluorescence. As a best practice, good expressor lines were maintained by germinating seeds on plates with a hygromycin selection for the transgene, and by screening seedlings for strong mCherry fluorescence signals in roots and leaves. Thus, while caution is always needed to avoid potential reporter expression artifacts and gene silencing, healthy plant lines were readily identified and maintained for multiple generations, unlike concerns reported for several other Ca^2+^ reporters with alternative designs (Ast et al., 2017; Waadt et al., 2017).

### Calibrating CGf Ratios Based on a Maximum Calcium Saturated Signal

The ability to monitor the ratio between GFP fluorescence and a baseline mCherry signal creates an opportunity to more reliably compare relative magnitudes of Ca^2+^ signals between different tissues, cell types, and within different subcellular locations. Without an internal baseline reference as provided here by the mCherry domain, it is not possible to know if different intensities from the GFP fluorescence are due to different amounts of reporter, or differences in [Ca^2+^]_cyt_. However, a caveat to using a ratiometric strategy is that the individual fluorescence intensities for both mCherry and GCaMP6f domains can vary under different imaging acquisition parameters, as occurs with adjustments of exposure times for each fluorophore, differences in excitation or emission wavelengths, or different background corrections appropriate for different tissues or conditions. Thus, it is important to first optimize imaging parameters and then apply those parameters across all experiments to reliably compare Ca^2+^ signals.

**FIGURE 4:**
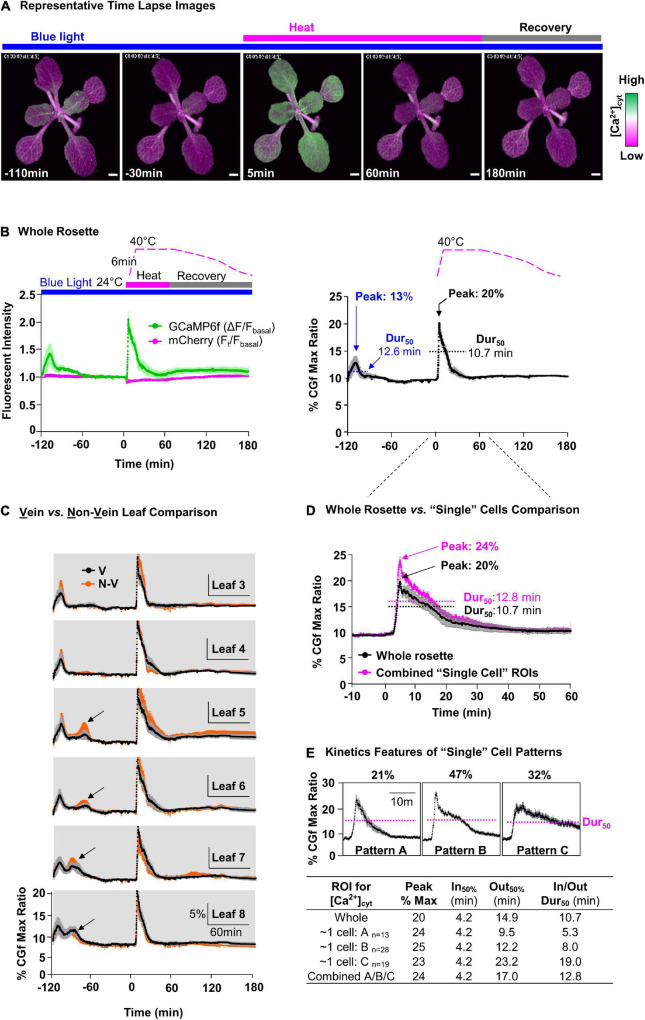
Comparative measurement of blue light- and heat-triggered Ca^2+^ changes in whole rosettes and “single cell”-sized areas of leaves. **(A)** Representative time lapse images of blue light and heat-triggered Ca^2+^ responses in plants expressing CGf. Magenta bar = heat stress. Gray bar = post-heat stress recovery. Blue bar indicates a continuous blue-light exposure (ex440/40 nm with 24 μmol m^– 2^s^– 1^ intensity). Scale bar = 1 mm. **(B)** Time series analysis of blue light and heat stress [Ca^2+^]_cyt_ responses in the rosette. ‘B, Left’ shows GCaMP6f (green) and mCherry (magenta) signals. Time course for heat treatment: 2 h pre-heat acclimation (–120 to 0 min), 1 h heat stress (0–60 min), and 2 h post-heat stress recovery periods (60–180 min). “B, Right” shows % of CGf max ratio. 50% durations (Dur_50_) were calculated as the time from the T_1/2_ of influx to the same concentration during efflux. The blue light peak is marked at –110 min and heat stress peak just after 0 min. **(C)** Time series analysis for leaves with a comparison of Ca^2+^ responses between vein (V, black) and non-vein (N-V, orange) tissues. Black arrows mark additional blue light-specific Ca^2+^peaks that are not present in the oldest leaves. Leaf numbers indicate order of development, from the oldest (Leaf 3) to the youngest (newest) detectable (Leaf 8). **(D)** Comparative analysis of heat-triggered Ca^2+^changes between whole rosette (black) and combined average of “single cell”-sized ROIs (magenta). Error bars are SEM of *n* = 5 independent plants (whole rosette) or *n* = 60 ROIs from the same set of 5 plants. **(E)** Characterization of three distinct heat-triggered Ca^2+^ patterns (A,B,C) in “single cell”-size ROIs with respective n, influx rate (In_50%_), and efflux rate (Out_50%_), and Dur_50_.

Another method to consider is normalizing ratio signals to a maximum ratio observed in a given tissue or cell type. For CGf expression in leaves, a 100% max signal was estimated by creating a wound site that disrupted the integrity of cellular Ca^2+^ stores and created a long-lived [Ca^2+^]_cyt_ increase that drove the ratiometric output to a maximum (Supplementary Figure 3). Using this approach, the heat-triggered Ca^2+^ signals described below were estimated to be approximately 20% of the maximum saturated potential for the CGf reporter (Figure 4). However, caution is required to not over-interpret the precision of estimating true *in vivo* concentrations, as unknown differences between *in vivo* and *in vitro* conditions can dramatically alter calibrations. Regardless, a peak signal of 20% of CGf’s maximum still represents more than a 2-fold relative increase in [Ca^2+^]_cyt_ over unstimulated resting levels.

### Heat-Triggered Cytosolic Calcium Signals Throughout the Rosette

To visualize heat-triggered Ca^2+^ signals in rosette leaves, seedlings were first adapted to dim-light to ensure a reproducible starting point for imaging. The imaging time course was then initiated by the exposure of plants to blue light (Ex470/40 nm with 24 μmol m^–2^s^–1^ intensity) to excite the GFP domain of CGf (Figures 4A,B, and left). This initial blue light stimulation reliably triggered Ca^2+^ signals that reached an average magnitude around 13% of CGf’s maximum ratio, with 50% durations measured around 12.6 min (Figure 4B, right, and Supplementary Movie 1). A 50% signal duration was calculated starting at the time Ca^2+^ influx reached 50% of its maximum peak.

The heat stress was initiated 2-h after the start of imaging to ensure that plants had recovered from the initial blue light exposure and other unintended stimuli. The heat stress was introduced using a heating pad inside an enclosure (Supplementary Figure 4). The temperatures ramped from ∼ 24 to 40^°^C over a short 6 min period. However, even before reaching 40^°^C, the changing temperatures triggered a rapid and steady rise in [Ca^2+^]_cyt_ with a peak corresponding to 20% of CGfs maximum ratio, which was approximately 1.5-fold higher than the blue light triggered signals at the start of the imaging experiments. While the 40^°^C temperature was maintained for a full hour, the peak [Ca^2+^]_cyt_ immediately began a relatively slow decline to produce a transient with a 50% duration of 10.7 min, which was slightly faster than the 12.6 min for blue light-triggered signal (Figure 4B, right).

### Heat Stress [Ca^2+^]_cyt_ Signals Occur With Similar Kinetics in All Rosette Leaves

To evaluate whether there were differences between the heat stress responses in different leaves, whole plant responses were reanalyzed at the level of individual leaves. The signal traces were grouped according to leaf age from the oldest (Leaf 3, first detectable true leaf) to the newest (e.g., Leaf 8) (Figure 4C). This analysis indicated that all leaves in a rosette showed a high degree of similarity for their heat sensing threshold, as well as their peak magnitudes and 50% signal durations. In addition, very similar kinetic profiles were observed for signals corresponding to veins and non-vein regions of the leaves. However, a leaf-age dependent variation was observed for the initial blue light signals, with the youngest (newest) leaves showing an additional second peak (see newer leaves L5, L6, L7, L8, in Figure 4C).

### Heat Stress [Ca^2+^]_cyt_ Signals in Leaves Occurred With Three Different Kinetic Patterns

To evaluate whether there were signaling differences at the “single cell” level, images were also analyzed with smaller regions of interest (ROI)s in non-vein tissues (Figure 4C). While the ROIs were approximately the surface area of a single pavement cell, the signals collected from these ROIs actually represented several cells because the fluorescence contributed from the underlying cell layers. For the “single cell” ROIs selected, the summation of their combined signal traces was very similar to the average for the whole rosette (Figure 4D). However, in contrast to whole rosette or individual leaf analyses (Figures 4B,C), signals from these “single cell” ROIs showed variations that could be clustered into three patterns with distinct kinetic features (Figure 4E). A key kinetic difference was in the 50% durations, which were 5.3, 8.0, and 19 min, respectively (Figure 4E).

### CGf Detected Two Types of Ca^2+^ Oscillations in Pollen Tubes Under Normal Conditions

**FIGURE 5:**
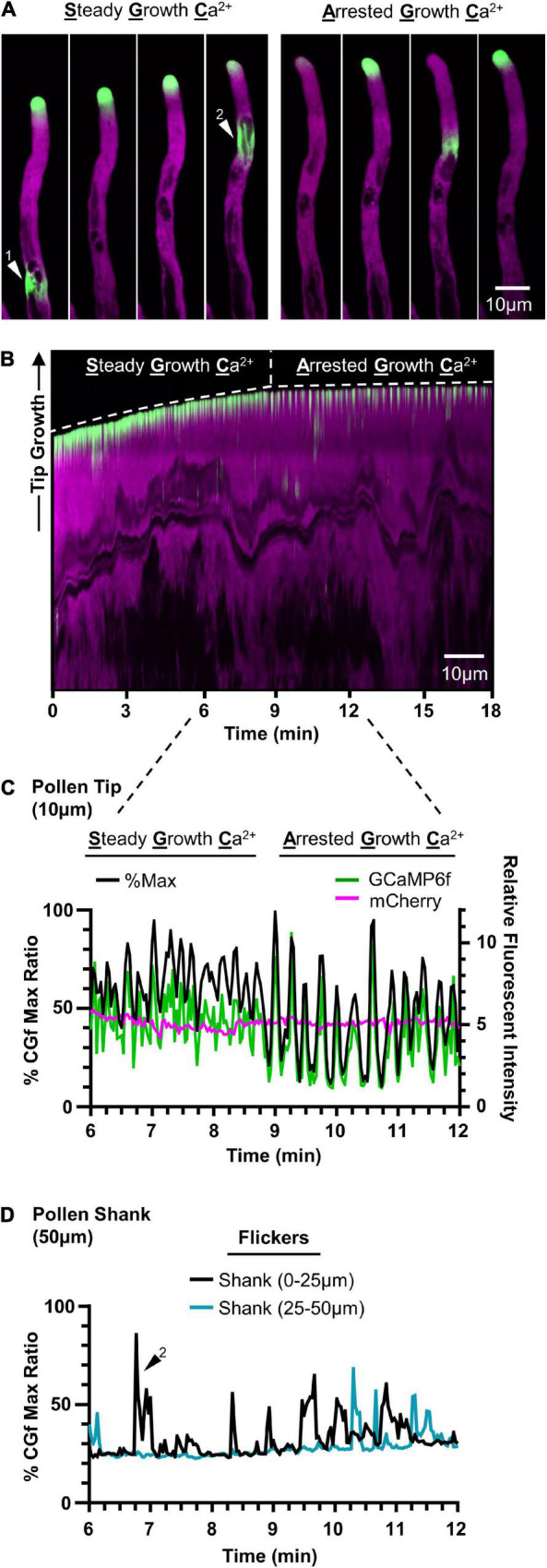
CGf detects Ca^2+^ signals associated with steady growth, growth arrest, and flickers in *Arabidopsis* pollen tubes. Representative time lapse images **(A)** and kymograph **(B)** showing steady growth tip-focused Ca^2+^ (SGC) oscillations, arrested growth Ca^2+^ (AGC) oscillations, and Ca^2+^ flickers in the pollen shank within the same pollen tube over 18 min. Example flickers are marked by white triangles 1 and 2. **(C)** Comparison of pollen tip oscillatory patterns and magnitudes between SGC and AGC oscillations using CHUKNORRIS (Damineli et al., 2017). Relative fluorescent intensities (right) of GCaMP6f (green) and mCherry (magenta) with corresponding % of CGf max (black, left). Tip = first 10 μm. **(D)** Ca^2+^ flickers display similar magnitudes to tip-focused ratios, as shown by representative flicker 2, and are observed as localized changes scattered throughout the pollen shank. Shank = 50 μm following the 10 μm tip region.

In evaluating CGf’s ability to detect subcellular Ca^2+^ signals in growing pollen tubes, two patterns of tip-focused [Ca^2+^]_cyt_ oscillations were observed (Figure 5 and Supplementary Movies 3, 4), similar to those previously reported using YC3.6 (*K*_D_ 250 nM) (Damineli et al., 2017) and GCaMP5 (*K*_D_ 450 nM) (Akerboom et al., 2012). These two patterns are referred to here as steady growth Ca^2+^ (SGC) oscillations and arrested growth Ca^2+^ (AGC) oscillations. Time lapse images and a representative kymograph show a transition from SGC to AGC oscillations within the same pollen tube over a 18 min imaging window at room temperature (Figures 5A,B and Supplementary Movie 3). SGC oscillations were rapid, shallow oscillations with a high baseline average [Ca^2+^]_cyt_, while AGC oscillations showed peaks with higher magnitudes and clear intervening periods of very low resting baseline [Ca^2+^]_cyt_ (Figure 5C).

It appeared that tip-focused Ca^2+^ oscillations often displayed peak signal intensities that ranged from 70 to 100% of the Ca^2+^ saturated maximum. While these high signal ratios were outside the linear calibration range for CGf, they suggest that many signals reached magnitudes equal or greater than 960 nM [Ca^2+^]_cyt_, which corresponds to CGf’s estimated Ca^2+^ saturated maximum.

In addition to confirming two common patterns of tip-focused Ca^2+^ oscillations, rapid [Ca^2+^]_cyt_ transients of equal magnitude were also observed at dispersed locations in the pollen tube shank, as shown in time lapse images (Figure 5A) and time series analysis (Figure 5D). Described here as flickers, these signals often showed 50% durations as rapid as 0.5 s, suggesting these signals could easily be missed if intervals between image captures are longer than 2 s. Flickers did not appear to be synchronized with tip oscillations, nor necessarily show repeating oscillations at the same subcellular locations (Supplementary Movie 4). While a survey of the literature indicates that flickers are often observed in time lapse images (Keinath et al., 2015; Diao et al., 2018), there has been little discussion about their kinetic features or speculation about their biological meaning.

### *Arabidopsis* Pollen Responds to Temperature Increases by Dampening Tip-Focused Ca^2+^ Dynamics

**FIGURE 6:**
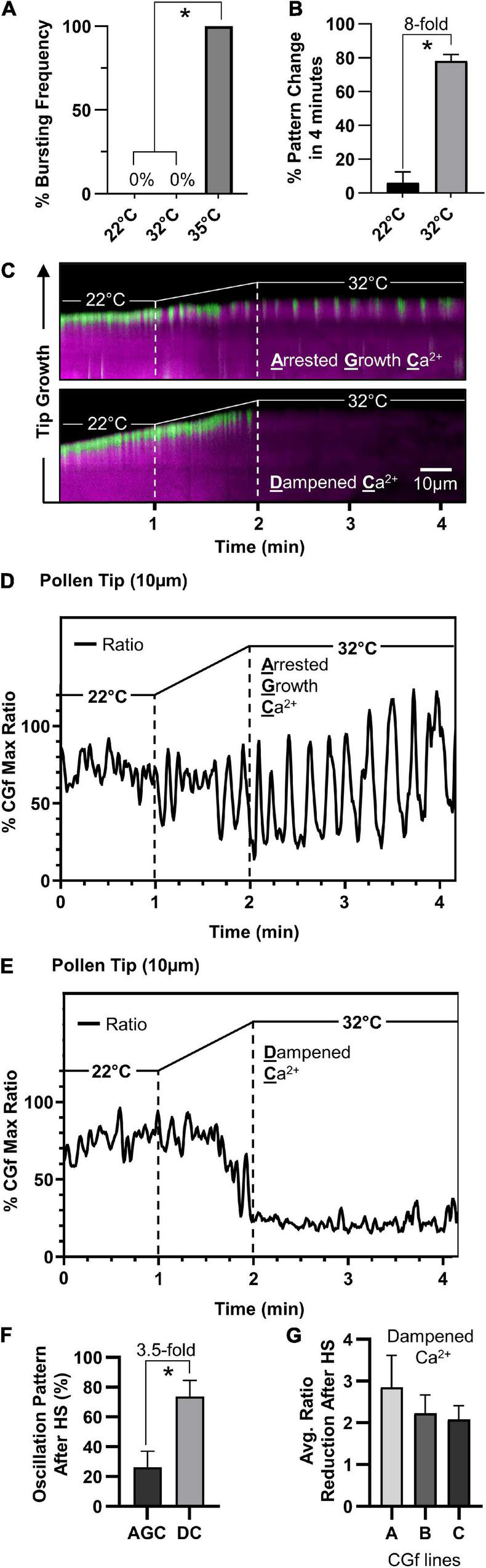
*Arabidopsis* pollen respond to temperature increases by attenuating tip-localized Ca^2+^ dynamics. **(A)** Pollen tubes did not rupture at 32°C compared to 100% bursting at 35°C (*n* = 76, *p* < 0.001). **(B)** 32°C heat stress results in significantly more shifts in tip-focused oscillation patterns compared to 22°C controls (*n* = 51, *p* < 0.001). **(C)** Kymograph representation of steady growth tip-focused Ca^2+^ (SGC) oscillations shifting to one of two patterns under heat stress, an arrested growth Ca^2+^ (AGC) oscillation or a dampened Ca^2+^ (DC) oscillation. Time course: 22°C for 1 min, 22–32°C in 1 min, followed by 2 min at 32°C. **(D,E)** CHUKNORRIS time series analysis of tip-focused [Ca^2+^]_cyt_ shifting from SGC oscillations to **(D)** heat-triggered AGC oscillations or **(E)** heat-triggered DC oscillations. CGf Ratios are shown as a% of Max. **(F)** At 32°C, DC oscillations are observed 3.5 times more often than AGC oscillations (*n* = 30, *p* < 0.01*).* **(G)** The average tip-focused [Ca^2+^]_cyt_ during SGC oscillations is decreased by more than twofold in all three reporter lines analyzed. Error bars are SEM of *n* = 3 pollen tubes for each independent line (CGf line A ss2540, line B ss2640, and line C ss2641). * = statistical significance for each respective panel.

To investigate the effects of heat stress on calcium dynamics in pollen tubes, we imaged and analyzed pollen tubes showing **s**teady growth tip-focused **C**a^2+^ (SGC) oscillations prior to the heat exposure. Considering that a 35°C heat stress increased bursting frequency 100-fold, a milder heat stress exposure was applied by increasing the temperature from 22 to 32°C (1°C per 6 s for 1 min) (Figure 6A), a stress temperature also used in other studies (Muhlemann et al., 2018; Luria et al., 2019). In this study, a 32°C heat stress increased the frequency of a Ca^2+^-pattern shift by eightfold (Figure 6B).

Imaging with CGf revealed that SGC oscillations shifted to one of two patterns under heat stress, an **a**rrested **g**rowth **C**a^2+^ (AGC) or a **d**ampened **C**a^2+^ (DC) oscillation, as shown in representative kymographs (Figure 6C). Time series analysis revealed heat-triggered AGC oscillations (Figure 6D and Supplementary Movie 5) appeared to have similar kinetic features to those that were occasionally observed under non-stress conditions (Figure 5C), as characterized by high magnitudes and clear intervening periods of very low resting baseline [Ca^2+^]_cyt_. DC oscillations were characterized by very shallow oscillations on top of an almost undetectable baseline [Ca^2+^]_cyt_ (Figure 6E and Supplementary Movie 6). Additionally, DC oscillations were observed 3.5-fold more often than AGC oscillations (Figure 6F). The average [Ca^2+^]_cyt_ associated with DC oscillations was reduced by at least 50% compared to pre-stressed SGC oscillations, as confirmed in all three independent transgenic lines evaluated (Figure 6G). To ensure that heat-triggered dampening of tip-focused [Ca^2+^]_cyt_ was not an artifact of cell death, heat-stressed pollen tubes were allowed to recover and imaged thereafter. In all cases pollen tubes remained viable, as indicated by growth or the appearance of [Ca^2+^]_cyt_ oscillations. For a subset of tubes, further imaging was conducted after a 1 h recovery period. For the 6 tubes that showed a DC oscillation pattern during heat stress, after a 1 h recovery period they all showed a restoration of [Ca^2+^]_cyt_ oscillations (e.g., Supplementary Movie 7) and growth rates between 0.25 and 2.6 μm/min, which overlaps with growth rates for unstressed-pollen tubes grown in parallel (Supplementary Figure 6).

In contrast to heat-triggered signals observed in leaves (Figure 4), we failed to see any evidence for a sustained heat-triggered increase in [Ca^2+^]_cyt_ in the pollen tube shank (Figure 6C).

## Discussion

The CGf Ca^2+^ reporter developed here was used to conduct ratiometric imaging of heat stress triggered [Ca^2+^]_cyt_ dynamics in whole seedlings, leaves, “single cell”-sized regions of interest, and single cell pollen with subcellular resolution (Figures 4, 6). The reporter’s design provides a stable mCherry-red fluorescence as a reference for normalizing dynamic changes in green fluorescence caused by Ca^2+^ interactions with the GCaMP6f domain (Figure 1). Without a normalization control for GCaMP6f, it is difficult to distinguish between real Ca^2+^ dependent changes and artifacts of reporter abundance in a particular leaf, cell type, or subcellular region. Here we used the ratiometric features of CGf to identify five different patterns of heat-triggered modifications to Ca^2+^ dynamics in *Arabidopsis*.

### The Expanding Palate of Ca^2+^ Reporters

CGf represents a new addition to a growing diversity of Ca^2+^ reporters, such as the YCnano65 (Horikawa et al., 2010), Matrosky (Ast et al., 2017), and R-GECO1-mTurquoise (Waadt et al., 2017). Each of these reporters has advantages for different imaging equipment and specific biological applications. A reporter that most closely resembles CGf was similarly designed as a fusion of GCaMP6f with an mCherry, but differs in that its mCherry domain was fused to the C-terminal instead of N-terminal end of GCaMP6f (Waadt et al., 2017). A concern raised about this previous design was its potential to cause growth deficiencies. While stable overexpression of any reporter has potential for negative impacts, the frequency of independent transgenic lines showing an obvious phenotypes for CGf was estimated here at less than 1 in 10, with normal growth and reproduction confirmed by quantification of rosette sizes, root growth rates, seed numbers per silique, total seed set, and reciprocal crosses to test for normal Mendelian segregation of the transgene (Figure 5 and Supplementary Figure 2).

The fusion of an mCherry to GCaMP6f did not appear to significantly alter GCaMP6f’s Ca^2+^ affinity, as indicated by a side-by-side Ca^2+^ titration comparison with a single fluorescent reporter (Figure 2). An apparent *K*_D_ of 220 nM was estimated for CGf, which represents an overlap between the ranges measured here for CGf and GCaMP6f (201–223 nM, Figure 2) and the low end of the 220–375 nM range reported in the literature for GCaMP6f (Helassa et al., 2016; Chen et al., 2013).

CGf’s ratiometric design represents an important feature that permits different Ca^2+^ signals to be compared for relative differences in peak magnitudes and signal durations. The various signal intensities observed here were calculated as a percentage of CGf’s Ca^2+^ saturated maximum based on imaging conditions used in this study. The heat stress signals in leaves were estimated at around 20–25% of the CGf maximum (Figure 4), whereas signals observed in pollen often reached magnitudes close to 100% (Figure 5). With the caveat that *in vitro* calibration curves cannot precisely predict an *in vivo* [Ca^2+^]_cyt_, a 50% maximum signal *in planta* will likely be close to the reporters *K*_D_ concentration (Figure 2). Thus, the heat stress signals observed in leaves showed magnitudes that appeared to be less than CGfs *K*_D_ around 220 nM, whereas signals in pollen showed magnitudes that likely rose to near or above the estimated 960 nM needed to reach CGf’s maximum.

### Heat Stress Triggers Multiple Ca^2+^ Signals in Leaves

CGf-based analyses in leaves provide strong evidence that heat stress can induce cytosolic Ca^2+^ signals in plants (Figure 4). Prior studies with aequorin provided mixed results (Gao et al., 2012; Finka and Goloubinoff, 2014; Lenzoni and Knight, 2019). In a recent study using aequorin, a heat-triggered Ca^2+^ signal was identified inside chloroplasts, but not in the cytoplasm, using young cotyledon staged seedlings (Lenzoni and Knight, 2019). Another aequorin study with *Arabidopsis* seedlings reported a gradual heat-dependent increase in [Ca^2+^]_cyt_, but this analysis was not extended for a sufficient period of time to confirm that [Ca^2+^]_cyt_ returned to a baseline resting level (Gao et al., 2012), as expected for a stereotypical Ca^2+^ signal. In *Physcomitrella*, heat stress also failed to induce a typical Ca^2+^ transient in wild type cells, although an increase in [Ca^2+^]_cyt_ was observed for a heat-sensitive mutant with a deletion of a cyclic nucleotide gated channel (Finka and Goloubinoff, 2014). These mixed results might be explained by aequorin’s relatively weak affinity for Ca^2+^ (*K*_D_ ∼ 7–13 μM) (Costa et al., 2018), which makes it a suboptimal reporter for detecting the types of low nM [Ca^2+^] signals reported here using a CGf (*K*_D_ ∼ 220 nM). In addition, the aequorin reporter requires the addition of a substrate. Therefore, mixed results may be due to different efficiencies of substrate loading or other constraints on substrate/aequorin interactions.

The CGf reporter also showed that heat-triggered signals were approximately 1.5-fold greater in magnitude than the blue light signals in the same leaves (Figure 4). Blue light signals were previously documented using aequorin (Harada et al., 2003), YCnano65, and a GCaMP6 (Ishka et al., 2021). Evidence indicates that blue light activates phototropin receptors that trigger a Ca^2+^ induced Ca^2+^ release from internal Ca^2+^ stores (Harada and Shimazaki, 2007). Interestingly, there is also evidence that phototropins contribute to temperature perception (Hayes et al., 2021). However, they are reported to have increased activity at lower temperatures, which argues that they are unlikely candidates for mediating a heat-induced Ca^2+^ influx. In addition, the kinetic differences seen here between blue light and heat-triggered signals suggests that the cellular machinery involved in generating each of these signals is either different or subject to different regulatory controls. While several candidate channels for heat-triggered Ca^2+^ entry have been proposed, such as cyclic nucleotide gated channels (Tunc-Ozdemir et al., 2013; Wang et al., 2021), it is not yet clear which channels might actually contribute to the heat stress signatures identified here in leaves.

### Heat Stress Suppresses Normal Growth Associated Ca^2+^ Signals in Pollen

The CGf analyses with pollen provided an example of a heat stress response that appears very different than leaves. Unlike leaves, pollen tubes failed to show a heat-triggered increase in [Ca^2+^]_cyt_, either at the tip or elsewhere in the tube shank (Figure 6). Instead, the normal steady growth Ca^2+^ oscillations at the pollen tube tip shifted to a new oscillation pattern typical of a growth arrest (22% of cases) or a severely dampened oscillation with a nearly undetectable baseline [Ca^2+^]_cyt_ (78% of cases).

While pollen fertility is considered highly vulnerable to heat stress, the underlying causes remain speculative and are likely different during various phases of pollen development and fertilization (Johnson et al., 2019). It is noteworthy that experiments here revealed that a relatively small increase in the maximum heat from 32 to 35°C was accompanied by a more than 100-fold increase in the frequency of pollen tube tip ruptures (Figure 6A). This suggests that rapid tip growth processes represent a point of thermo-vulnerability, possibly because heat stress disrupts the precise coordination required to stabilize newly delivered membranes and cell wall structures at the growing tip. Regardless, the observed heat stress suppression of tip-focused [Ca^2+^]_cyt_ oscillations occurred within a minute, correlating with a rapid and potentially thermo-protective switch to a pause in tip growth.

### Using Ratiometric Reporters to Catalog the Diversity of Stimulus-Specific Ca^2+^ Signals in Plants

CGf and other ratiometric reporters are often brighter and provide stronger signals than FRET-based sensors such as YCnano65. Thus, CGf-like reporters represent an important experimental opportunity to compare and classify different stimulus-specific Ca^2+^ signals. While it is not yet clear how many functionally different Ca^2+^ signals are generated in plants, pollen cells alone express at least 36 potential Ca^2+^-permeable ion channels, along with multiple kinetic-modifying Ca^2+^ pumps and exchangers located in various compartments including the vacuole, ER, plasma membrane and other membrane organelles (Johnson et al., 2019). This complexity of cellular machinery creates an expectation for a large diversity of Ca^2+^ signals throughout the plant.

While three kinetically distinct heat-triggered [Ca^2+^]_cyt_ signatures were identified in leaves (Figure 4), it is likely that additional signals will be uncovered as more individual cell types are examined and different heat stress scenarios are considered. Importantly, heat stress in the real world is often accompanied by additional combinatorial stress factors, such as drought, high light, and nutritional limitations (Zandalinas et al., 2021), all of which are expected to uniquely impact the transcription and regulation of the machinery coding and decoding Ca^2+^ signals in different cells.

The current study suggests that both heat stress and blue light signals in vegetative cells occur through a rapid Ca^2+^_cyt_ influx followed by a relatively slow efflux, with 50% durations ranging from 5 to 19 min (Figure 4). In contrast, [Ca^2+^]_cyt_ signals in pollen were as much as 2,000-times faster, with 50% durations ranging from 0.5 to 5 s (Figure 5). These kinetic differences likely have profound consequences in the context of downstream signaling events. For example, a 5–19 min continuous elevation in [Ca^2+^]_cyt_ provides ample time for Ca^2+^ to diffuse throughout the cell and create long-lasting physiological changes, such as sustained activation of Ca^2+^-dependent phosphoregulatory networks and transcriptional changes leading to a long-term acclimation response (Liu et al., 2018; Alves et al., 2021; Damaris and Yang, 2021; Noman et al., 2021). In contrast, the rapid and highly localized [Ca^2+^]_cyt_ signals at the pollen tube tip are likely to have more restricted tip-focused functions related to rapid growth, such as regulating dynamics of secretion, actin filaments, and other components playing key roles in the tip-focused growth machinery (Qian and Xiang, 2019). The observation that heat stress suppresses these growth-associated [Ca^2+^]_cyt_ signals (Figure 6) supports a model in which the most urgent need for heat stress signaling in pollen tubes is to shift [Ca^2+^]_cyt_ oscillations at the growing tip into a growth arrest mode because a failure to do so could lead to an asynchronization of the growth machinery and an increased frequency of pollen tube ruptures.

These contrasting examples of heat-triggered [Ca^2+^]_cyt_ responses in leaves and pollen highlight the diversity of Ca^2+^ signals generated in plants, and more so, a need to better understand the underlying channels, transport systems, and signal transduction networks responsible for creating and decoding Ca^2+^ signals in plants.

## Data Availability Statement

The datasets presented in this study can be found in online repositories. The names of the repository/repositories and accession number(s) can be found in the article/Supplementary Material.

## Author Contributions

CW led the experiments on pollen imaging and helped design all experiments. S-HK conducted whole-plant imaging in response to heat stress and data analyses. EB conducted the *in vitro* spectrofluorometer studies. EM conducted effects of CGf expression to root, rosette, and seed sets development. MM conducted Western Blot analyses. GM and JH initiated the CGf reporter design for pollen. JH led the development of transgenic lines, reporter constructs, and genetic analyses. W-GC led the experiments on seedling imaging. W-GC and JH designed the experiments and supervised the overall project. All authors contributed constructive comments on the manuscript.

## Conflict of Interest

The authors declare that the research was conducted in the absence of any commercial or financial relationships that could be construed as a potential conflict of interest.

## Publisher’s Note

All claims expressed in this article are solely those of the authors and do not necessarily represent those of their affiliated organizations, or those of the publisher, the editors and the reviewers. Any product that may be evaluated in this article, or claim that may be made by its manufacturer, is not guaranteed or endorsed by the publisher.

## Footnotes

1
https://feijolab.shinyapps.io/CHUK/
