# Supplementary material for: A Ratiometric Calcium Reporter CGf Reveals Calcium Dynamics Both in the Single Cell and Whole Plant Levels Under Heat Stress
Source: Front Plant Sci. 2021 Dec 17;12:777975. doi: 10.3389/fpls.2021.777975 (PMC8718611; doi:10.3389/fpls.2021.777975)
Supplement: Supplementary file 1 [file Data_Sheet_1.PDF]

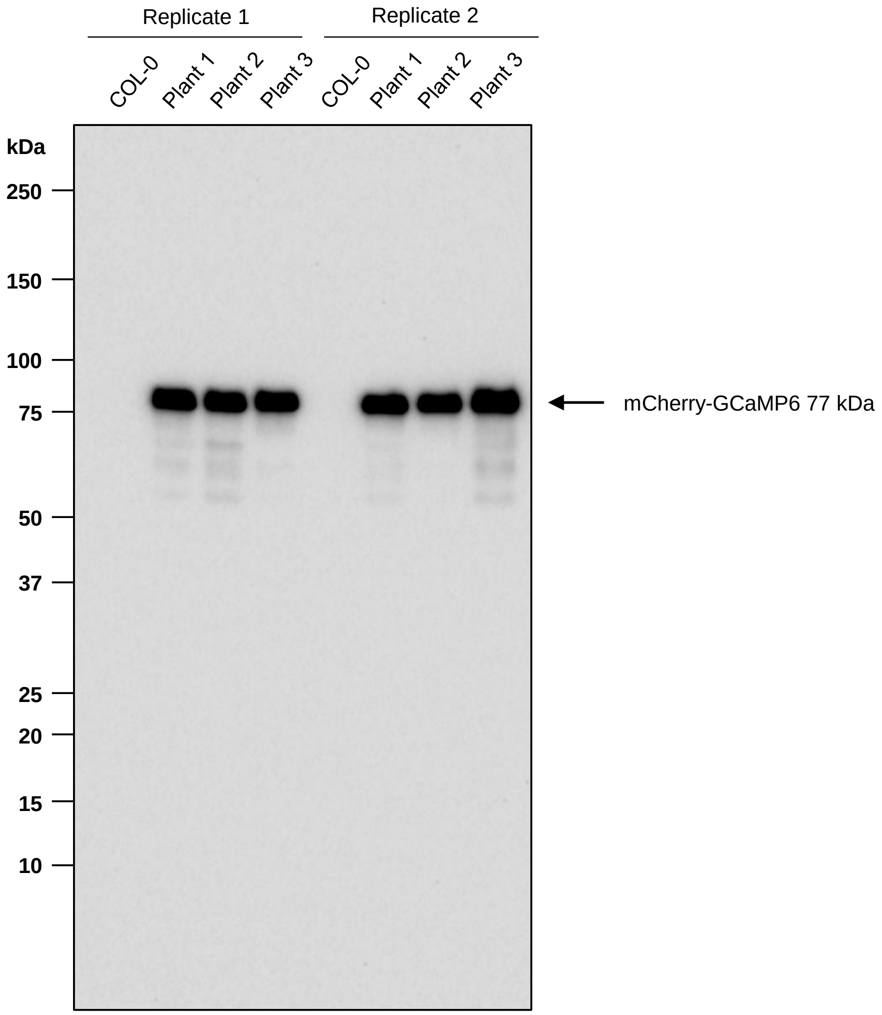

Supplemental Figure S1. Western blot detection of full length mCherry-GCaMP6 proteins (77kDa) *in planta* using primary RFP monoclonal antibody and secondary F(ab')2-Goat anti-Mouse IgG (H+L) (HRP) antibody. Left to right, Lane 1-8: COL-0 negative control, CGf, CGm, CGu, followed by confirmation with second transgenic line in lanes 5-8. CGf (fast), CGm (medium), and CGu (ultra) are identical m**C**herry fusions to three **G**CaMP6 variants GCaMP6**f** (K_D 220-375nM), GCaMP6**m** (K_D 167nM), and GCaMP6**u** (K_D 890nM) (Chen et al., 2013; Helassa et al., 2016).

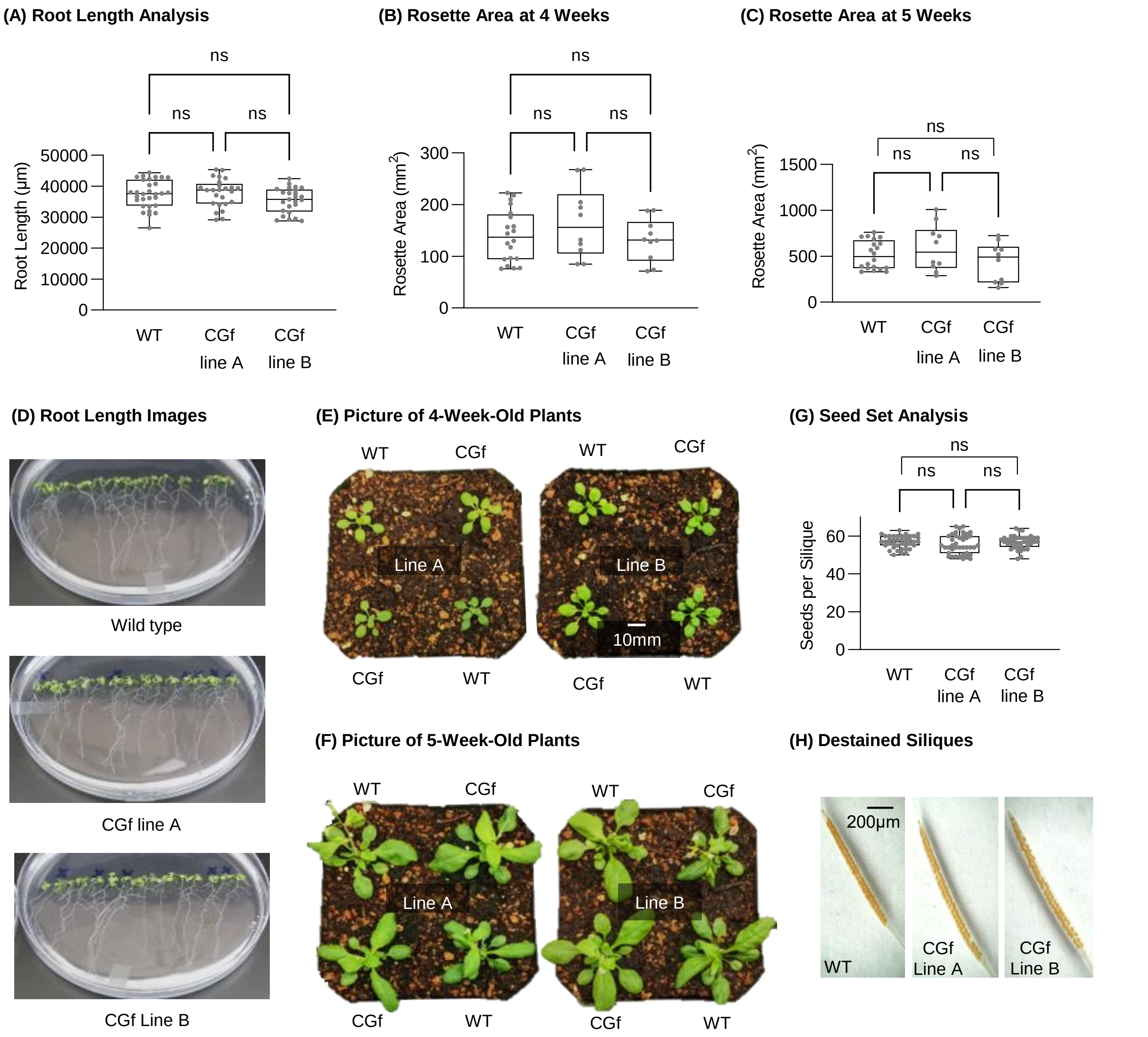

Supplemental Figure S2. Growth comparisons between wild type and CGf plants provide evidence that CGf expression did not alter plant development and reproduction. (A) Comparison of root lengths of 8-day old seedlings grown on plant media described in methods. n=30 (WT) or n=25 (CGf line A and B) from 5 biological replicates. **(B,C)** Measurement of total rosette area of the (B) 4-week-old and (C) 5-week-old plants. n=20 (WT) or n=10 (CGf line A and B) from five biological replicates. **(D- F)** Representative pictures of root length or plant growth shown in above data (A-C, respectively). **(G)** Seed set comparison between WT and two independent CGf lines. n=40 of siliques for all 3 groups from 10 independent plants in replicate. **(H)** Representative photos of decolorized siliques used to measure seed set. Scale bar = 200µm. **(A-C, G)** Statistical analyses were done using One-way ANOVA multiple comparisons with the Turkey post-hock test. Error bars in each box plot show the min to max values of representing data sets (grey dots). “ns” = not significant. All figures are comparisons between WT and two independent lines: CGf line A (ss2543) and line B (ss2544).

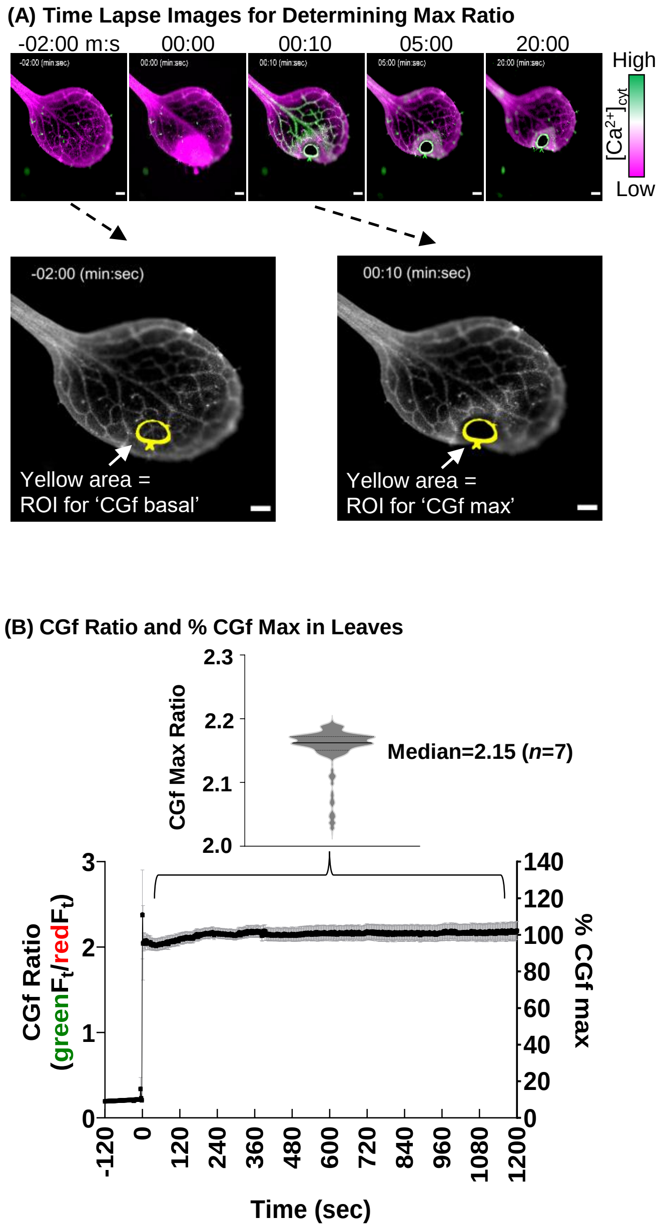

Supplemental Figure S3. Determining maximum CGf ratio in leaf. (A) Representative time lapse images of a true leaf expressing CGf. Time indicates minutes:seconds (m:s). Negative time points represent before stimulation, while positive time points are after burning a small leaf area for 2 seconds with a high power, red laser (Laser 301, 1000 MW 650nm Red Beam Light Single-point Laser Pointer; WDLaser Kowloon, Hong Kong) for 2 seconds. Color scale indicates $[Ca^{2+}]_{cyt}$ levels. Scale bar = 500μm. An enlarged representative black & white frame images show areas selected for detecting CGf basal (yellow ROI on the left side -2 min time point image) or CGf max (yellow ROI on the right side 10 sec image). **(B)** Fluorescent intensity and % CGf max of CGf signals in leaves at the laser-induced wound site. Fluorescent signal changes were calculated as described in methods. Images were obtained at 2 second intervals for 22min (2min pre-stimulus period followed by 20min post-stimulus) using the Zeiss AxioZoom V16 microscope described in methods. Error bars represent SEM of n=7 independent plants. % CGf max were calculated from the fluorescent intensity data using CGf max ratio (2.15). greenF_t and redF_t indicate GCaMP6f fluorescent intensity (greenF_t) and mCherry fluorescent intensity (redF_t) at the time of event. Violin graph (inset) shows median of maximum CGf ratio. CGf max ratio was calculated using data points from 10 to 1200 second duration (n=4172 data points from n=7 independent plants). Solid line = median value of data. Dotted lines = quartile of data.

(A) Picture of Whole Plant Heat Chamber **(B) Dome Lid Diagram**

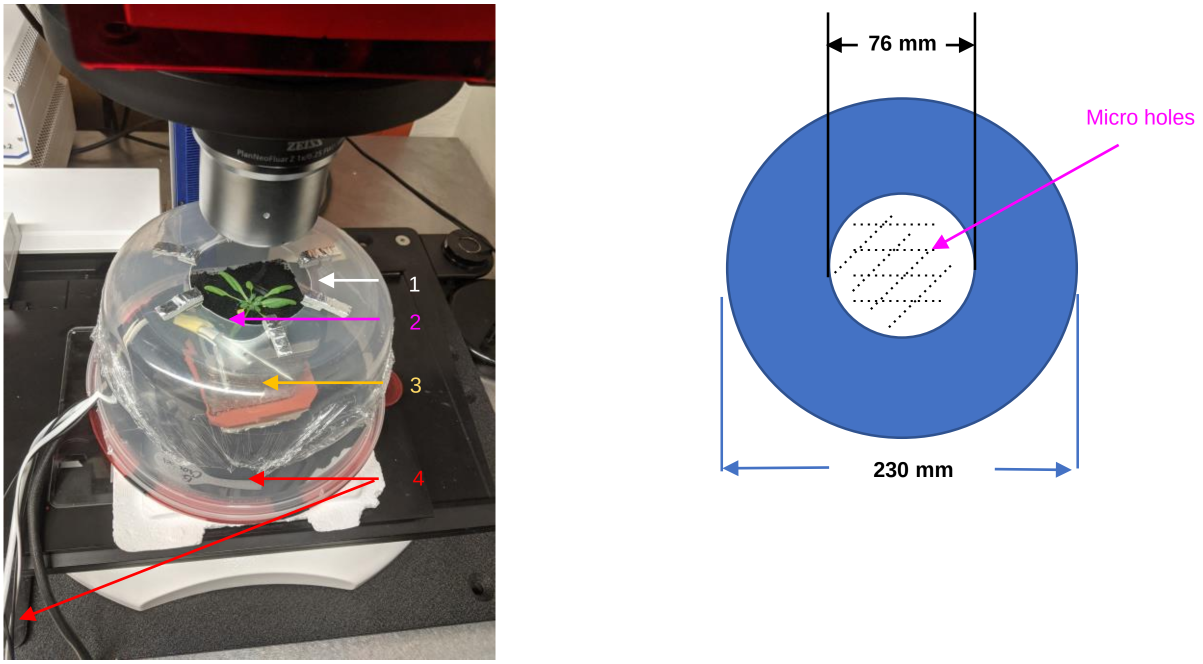

Supplemental Figure S4. Custom heat chamber used for whole plant heat stress experiments. (A) Photo of the custom heat chamber with a representative 5-week-old plants under a Zeiss AxioZoom V16 microscope. Picture legend as follows. 1) Dome lid of the heating chamber (white). 2) Representative 5-week-old WT plants grown in GA-7-3 magenta vessel. 3) 5mm thick silicon pad with heat insulation tape on the bottom (orange). A thick silicone pad with heat insulation tape is used to prevent direct heating of soil vessels during heat stress experiments. 4) A digital heating pad with a power cable (red). **(B)** Dome lid diagram showing open 76mm diameter window opening for imaging plants during heat stress experiments. To prevent drought during heat stress experiment, the 76mm diameter window was covered in clear plastic wrap with hundreds of micro-holes. Micro-holes were made using a 27-gauge needle to prevent condensation during heat stress experiment.

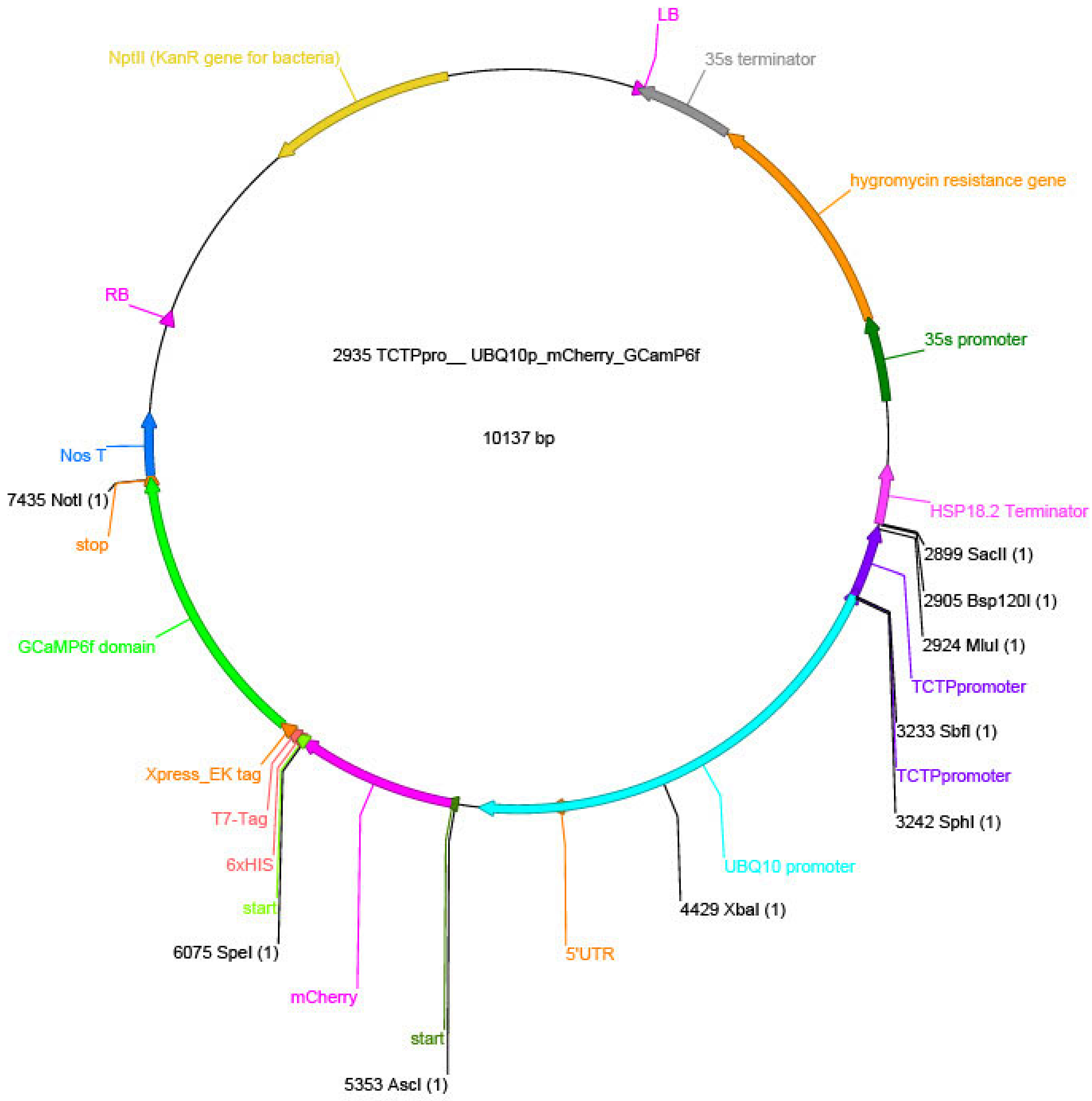

Supplemental Figure S5. CGf Plasmid Map and DNA Sequence

TCTPpro__ UBQ10p_mCherry_GCamP6f

1
TTTTTATCCCCGGAAGCCTGTGGATAGAGGGTAGTTATCCACGTGAAACCGCTAATGCCCCGCA
AAGCCTTGATTCACGGGGCTTTCCGGCCCGCTCCAAAAACTATCCACGTGAAATCGCTAATCAG
GGTACGTGAAATCGCTAATCGGAGTACGTGAAATCGCTAATAAGGTCACGTGAAATCGCTAATC
AAAAAGGCACGTGAGAACGCTAATAGCCCTTTCAGATCAACAGCTTGCAAACACCCCTCGCTCC
GGCAAGTAGTTACAGCAAGTAGTATGTTCAATTAGCTTTTCAATTATGAATATATATATCAATT
ATTGGTCGCCCTTGGCTTGTGGACAATGCGCTACGCGCACCGGCTCCGCCCGTGGACAACCGCA
AGCGGTTGCCCACCGTCGAGCGCCTTTGCCCACAACCCGGCGGCCGGCCGCAACAGATCGTTTT
ATAAATTTTTTTTTTTGAAAAAGAAAAAGCCCGAAAGGCGGCAACCTCTCGGGCTTCTGGATTT
CCGATCCCCGGAATTAGATCTTGGCAGGATATATTGTGGTGTAACGTTATCGATCTGGATTTTA
GTACTGGATTTTGGTTTTAGGAATTAGAAATTTTATTGATAGAAGTATTTTACAAATACAAATA
CATACTAAGGGTTTCTTATATGCTCAACACATGAGCGAAACCCTATAAGAACCCTAATTTCCCT
TATCGGGAAACTACTCACACATTATTTATGGAGAAAATAGAGAGAGATAGATTTGTAGAGAGAG
ACTGGTGATTTCAGCGTACCGAATTAATTCCTGAATCACTGCGACCGGCCCTCCCGCGACCCAG
CCGAGCGAGCTTAGCGAACTGTGGACGAGAACTGTGCCACCAAGCGTAAGGCCGTTCTCTCGCA
TTTGCCTTGCTAGGCTCGCGCGAGTTGCTGGCTGAGGCGTTCTCGAAATCAGCTCTTGTTCGGT
CGGCATCTACTCTATTCCTTTGCCCTCGGACGAGTGCTGGGGCGTCGGTTTCCACTATCGGCGA
GTACTTCTACACAGCCATCGGTCCAGACGGCCGCGCTTCTGCGGGCGATTTGTGTACGCCCGAC
AGTCCCGGCTCCGGATCGGACGATTGCGTCGCATCGACCCTGCGCCCAAGCTGCATCATCGAAA
TTGCCGTCAACCAAGCTCTGATAGAGTTGGTCAAGACCAATGCGGAGCATATACGCCCGGAGCC
TTGGCGATCCTGCAAGCTCCGGATGCCTCCGCTCGAAGTAGCGCGTCTGCTGCTCCATACAAGC
CAACCACGGCCTCCAGAAGAAGATGTTGGCGACCTCGTATTGGGAATCCCCGAACATCGCCTCG
CTCCAGTCAATGACCGCTGTTATGCGGCCATTGTCCGTCAGGACATTGTTGGAGCCGAAATCCG
CGTGCACGAGGTGCCGGACTTCGGGGCAGTCCTCGGCCCAAAGCATCAGCTCATCGAGAGCCTG
CGCGACGGACGCACTGACGGTGTCGTCCATCACAGTTTGCCAGTGATACACATGGGGATCAGCA
ATCGCGCATATGAAATCACGCCATGTAGTGTATTGACCGATTCCTTGCGGTCCGAATGGGCCGA
ACCCGCTCGTCTGGCTAAGATCGGCCGCAGCGATCGCATCCATAGCCTCCGCGACCGGCTGAAG
AACAGCGGGCAGTTCGGTTTCAGGCAGGTCTTGCAACGTGACACCCTGTGCACGGCGGGAGATG
CAATAGGTCAGGCTCTCGCTGAACTCCCCAATGTCAAGCACTTCCGGAATCGGGAGCGCGGCCG
ATGCAAAGTGCCGATAAACATAACGATCTTTGTAGAAACCATCGGCGCAGCTATTTACCCGCAG
GACATATCCACGCCCTCCTACATCGAAGCTGAAAGCACGAGATTCTTCGCCCTCCGAGAGCTGC
ATCAGGTCGGAGACGCTGTCGAACTTTTCGATCAGAAACTTCTCGACAGACGTCGCGGTGAGTT
CAGGCTTTTTCATAGGGGGGATCAGCTTGGGCTGTCCTCTCCAAATGAAATGAACTTCCTTATA
TAGAGGAAGGGTCTTGCGAAGGATAGTGGGATTGTGCGTCATCCCTTACGTCAGTGGAGATGTC
ACATCAATCCACTTGCTTTGAAGACGTGGTTGGAACGTCTTCTTTTTCCACGATGCTCCTCGTG
GGTGGGGGTCCATCTTTGGGACCACTGTCGGCAGAGGCATCTTGAATGATAGCCTTTCCTTTAT
CGCAATGATGGCATTTGTAGGAGCCACCTTCCTTTTCTACTGTCCTTTCGATGAAGTGACAGAT
AGCTGGGCAATGGAATCCGAGGAGGTTTCCCGAAATTACCCTTTGTTGAAAAGTCTCAATAGCC
CTTTGGTCTTCTGAGACTGTATCTTTGACATTTTTGGAGTAGGGGTACGATAACATTAACGCTT
ACAATTTCCATTCGCCATTCAGGCTGCGCAACTGTTGGGAAGGGCGATCGGTGCGGGCCTCTTC
GCTATTACGCCAGCTGGCGAAAGGGGGATGTGCTGCAAGGCGATTAAGTTGGGTAACGCCAGGG
TTTTCCCAGTCACGACGTTGTAAAACGACGGCCAGTGAGCGCGCGTAATACGACTCACTATAGG
GCGAATTGGGTACCGGAGAGGCGCCTTATCTTTAATCATATTCCATAGTCCATACCATAGCACA
TACAGTAGTTATATGCTGCAGAAGAGATCCAACAAAACATTCACAATGGATTATAGAAACATTT
GTTTATTCATTATAATGAGATCTTACATTCATTTAATATTAGAAAAAGCCACAAATTCATAACA

CAACAAGCCAAGAAAAAAACACAAACTTAAGCACACAAGCTTTTTATTTGACACACCAAATATT
TCATCTTCATCTTCATATCCGCGGGGGCCCGATATCGGGTCCCACGCGTGGTCGCTTATTGATT
GTTTTCTCTCTCCGATGGATGCGAATAAACGAAAATCGAAGGTTGCGGGAGAGAGACGCAGCGA
CGAGGGGATTCTGAAGTGAGGTCCTAAGTTTGGTATTTATAGTCGAAACTATAGCGTAGGGTTT
TCAGGTAAATTTACAATTCTCGGTAAATTACAATTTTGGGCTTTAACTGTTTCCAATTTTCTAT
TTTTTACCCTTTCCTTTTTTTTAAATAAAGGAAATAAATCTGAACCGACGCTTAATGAGCCGGG
TTTGGTCCAACGGGTGGGGATTCGAGTGTTGGCCTGCAGGCGCATGCATATGAGTCTAGCTCAA
CAGAGCTTTTAACCCAAATTGGTACAATAGAATACAACTTTAGATCATAATTCTCAAAAGAAAG
AGATTCCTTAGCTATTCTATCTGCCACTCCATTTCCTTCTCGGCTTGTATGCACAAGCATAAAA
TCCTCAAACTTGCTAAGTAGATACTTTATGTCTTGGATAATTGGATTGAGACTTGACAAGCATA
ACTTTCATGTAACCAAAGACACAAGTTGCTGAGAATCCACCTCAAAAATGATCTTCCTATAATT
GAATCGGGATAATGACAGCACAGCCCATCTAAGAGCCTCCACTTCTACTTCCAGCACGCTTCTT
ACTTTTACCACAGCTCTTGCACCTAACCATAACACCTTCCCTGTATGATCGCGAAGCACCCACC
CTAAGCCACATTTTAATCCTTCTGTTGGCCATGCCCCATCAAAGTTGCACTTAACCCAAGATTG
TGGTGGAGCTTCCCATGTTTCTCGTCTGTCCCGACGGTGTTGTGGTTGGTGCTTTCCTTACATT
CTGAGCCTCTTTCCTTCTAATCCACTCATCTGCATCTTCTTGTGTCCTTACTAATACCTCATTG
GTTCCAAATTCCCTCCCTTTAAGCACCAGCTCGTTTCTGTTCTTCCACAGCCTCCCAAGTATCC
AAGGGACTAAAGCCTCCACATTCTTCAGATCAGGATATTCTTGTTTAAGATGTTGAACTCTATG
GAGGTTTGTATGAACTGATGATCTAGGACCGGATAAGTTCCCTTCTTCATAGCGAACTTATTCA
AAGAATGTTTTGTGTATCATTCTTGTTACATTGTTATTAATGAAAAAATATTATTGGTCATTGG
ACTGAACACGAGTGTTAAATATGGACCAGGCCCCAAATAAGATCCATTGATATATGAATTAAAT
AACAAGAATAAATCGAGTCACCAAACCACTTGCCTTTTTTAACGAGACTTGTTCACCAACTTGA
TACAAAAGTCATTATCCTATGCAAATCAATAATCATACAAAAATATCCAATAACACTAAAAAAT
TAAAAGAAATGGATAATTTCACAATATGTTATACGATAAAGAAGTTACTTTTCCAAGAAATTCA
CTGATTTTATAAGCCCACTTGCATTAGATAAATGGCAAAAAAAAACAAAAAGGAAAAGAAATAA
AGCACGAAGAATTCTAGAAAATACGAAATACGCTTCAATGCAGTGGGACCCACGGTTCAATTAT
TGCCAATTTTCAGCTCCACCGTATATTTAAAAAATAAAACGATAATGCTAAAAAAATATAAATC
GTAACGATCGTTAAATCTCAACGGCTGGATCTTATGACGACCGTTAGAAATTGTGGTTGTCGAC
GAGTCAGTAATAAACGGCGTCAAAGTGGTTGCAGCCGGCACACACGAGTCGTGTTTATCAACTC
AAAGCACAAATACTTTTCCTCAACCTAAAAATAAGGCAATTAGCCAAAAACAACTTTGCGTGTA
AACAACGCTCAATACACGTGTCATTTTATTATTAGCTATTGCTTCACCGCCTTAGCTTTCTCGT
GACCTAGTCGTCCTCGTCTTTTCTTCTTCTTCTATAAAACAATACCCAAAGAGCTCTTCTT
CTTCACAATTCAGATTTCAATTTCTCAAAATCTTAAAAACTTTCTCTCAATTCTCTCTACCGTG
ATCAAGGTAAATTTCTGTGTTCCTTATTCTCTCAAAATCTTCGATTTTGTTTTCGTTCGATCCC
AATTTCGTATATGTTCTTTGGTTTAGATTCTGTTAATCTTAGATCGAAGACGATTTTCTGGGTT
TGATCGTTAGATATCATCTTAATTCTCGATTAGGGTTTCATAGATATCATCCGATTTGTTCAAA
TAATTTGAGTTTTGTCGAATAATTACTCTTCGATTTGTGATTTCTATCTAGATCTGGTGTTAGT
TTCTAGTTTGTGCGATCGAATTTGTCGATTAATCTGAGTTTTTCTGATTAACAGGGATCATCAA
CAAGTTTGTACAAAAAAGCAGGCTCTTTAAAGTATTTTTACAACAATTACCAACAACAACAAAC
AACAAACAACATTACAATTACTATTTACAATTACACTCGAGGCGCGCCAGGTATGGtgagcaag
ggcgaggaggataacatggccatcatcaaggagttcatgcgcttcaaggtgcacatggagggct
ccgtgaacggccacgagttcgagatcgagggcgagggcgagggccgcccctacgagggcaccca
gaccgccaagctgaaggtgaccaagggtggccccctgcccttcgcctgggacatcctgtcccct
cagttcatgtacggctccaaggcctacgtgaagcaccccgccgacatccccgactacttgaagc
tgtccttccccgagggcttcaagtgggagcgcgtgatgaacttcgaggacggcggcgtggtgac
cgtgacccaggactccTCCCTACAGgacggcgagttcatctacaaggtgaagctgcgcggcacc
aacttcccctccgacggccccgtaatgcagaagaagaccatgggctgggaggcctcctccgagc

ggatgtaccccgaggacggcgccctgaagggcgagatcaagcagaggctgaagctgaaggacgg
cggccactacgacgctgaggtcaagaccacctacaaggccaagaagcccgtgcagctgcccggc
gcctacaacgtcaacatcaagttggacatcacctcccacaacgaggactacaccatcgtggaac
agtacgaacgcgccgagggccgccactccaccggcggcatggacgagctgtacaagGGACTAGT
Tatgggttctcatcatcatcatcatcatggtatggctagcatgactggtggacagcaaatgggt
cgggatctgtacgacgatgacgataaggatctcgccaccATGGTCGACTCATCACGTCGTAAGT
GGAATAAGACAGGTCACGCAGTCAGAGCTATAGGTCGGCTGAGCTCACTCGAGAACGTCTATAT
CAAGGCCGACAAGCAGAAGAACGGCATCAAGGCGAACTTCAAGATCCGCCACAACATCGAGGAC
GGCGGCGTGCAGCTCGCCTACCACTACCAGCAGAACACCCCCATCGGCGACGGCCCCGTGCTGC
TGCCCGACAACCACTACCTGAGCGTGCAGTCCAAACTTTCGAAAGACCCCAACGAGAAGCGCGA
TCACATGGTCCTGCTGGAGTTCGTGACCGCCGCCGGGATCACTCTCGGCATGGACGAGCTGTAC
AAGGGCGGTACCGGAGGGAGCATGGTGAGCAAGGGCGAGGAGCTGTTCACCGGGGTGGTGCCCA
TCCTGGTCGAGCTGGACGGCGACGTAAACGGCCACAAGTTCAGCGTGTCCGGCGAGGGTGAGGG
CGATGCCACCTACGGCAAGCTGACCCTGAAGTTCATCTGCACCACCGGCAAGCTGCCCGTGCCC
TGGCCCACCCTCGTGACCACCCTGACCTACGGCGTGCAGTGCTTCAGCCGCTACCCCGACCACA
TGAAGCAGCACGACTTCTTCAAGTCCGCCATGCCCGAAGGCTACATCCAGGAGCGCACCATCTT
CTTCAAGGACGACGGCAACTACAAGACCCGCGCCGAGGTGAAGTTCGAGGGCGACACCCTGGTG
AACCGCATCGAGCTGAAGGGCATCGACTTCAAGGAGGACGGCAACATCCTGGGGCACAAGCTGG
AGTACAACCTGCCGGACCAACTGACTGAAGAGCAGATCGCAGAATTTAAAGAGGAATTCTCCCT
ATTTGACAAGGACGGGGATGGGACAATAACAACCAAGGAGCTGGGGACGGTGATGCGGTCTCTG
GGGCAGAACCCCACAGAAGCAGAGCTGCAGGACATGATCAATGAAGTAGATGCCGACGGTGACG
GCACAATCGACTTCCCTGAGTTCCTGACAATGATGGCAAGAAAAATGAAATACAGGGACACGGA
AGAAGAAATTAGAGAAGCGTTCGGTGTGTTTGATAAGGATGGCAATGGCTACATCAGTGCAGCA
GAGCTTCGCCACGTGATGACAAACCTTGGAGAGAAGTTAACAGATGAAGAGGTTGATGAAATGA
TCAGGGAAGCAGACATCGATGGGGATGGTCAGGTAAACTACGAAGAGTTTGTACAAATGATGAC
AGCGAAGTGAGCGGCCGCGAGCTCGAATTTCCCCGATCGTTCAAACATTTGGCAATAAAGTTTC
TTAAGATTGAATCCTGTTGCCGGTCTTGCGATGATTATCATATAATTTCTGTTGAATTACGTTA
AGCATGTAATAATTAACATGTAATGCATGACGTTATTTATGAGATGGGTTTTTATGATTAGAGT
CCCGCAATTATACATTTAATACGCGATAGAAAACAAAATATAGCGCGCAAACTAGGATAAATTA
TCGCGCGCGGTGTCATCTATGTTACTAGATCGGGAATTCAGCTCCAGCTTTTGTTCCCTTTAGT
GAGGGTTAATTGCGCGCTTGGCGTAATCATGGTCATAGCTGTTTCCTGTGTGAAATTGTTATCC
GCTCACAATTCCACACAACATACGAGCCGGAAGCATAAAGTGTAAAGCCTGGGGTGCCTAATGA
GTGAGCTAACTCACATTAATTGCGTTGCGCTCACTGCCCGCTTTCCAGTCGGGAAACCTGTCGT
GCCAGCTGCATTAATGAATCGGCCAACGCGCGGGGAGAGGCGGTTTGCGTATTGGGCGCTCTTC
CGCTTCCTCGCTCACTGACTCGCTGCGCTCGGTCGTTCGGCTGCGGCGAGCGGTATCAGCTCAC
TCAAAGGCGGTAATACGGTTATCCACAGAATCAGGGGATAACGCAGGAAAGAACATGAAGGCCT
TGACAGGATATATTGGCGGGTAAACTAAGTCGCTGTATGTGTTTGTTTGAGATCTCATGTGAGC
AAAAGGCCAGCAAAAGGCCAGGAACCGTAAAAAGGCCGCGTTGCTGGCGTTTTTCCATAGGCTC
CGCCCCCCTGACGAGCATCACAAAAATCGACGCTCAAGTCAGAGGTGGCGAAACCCGACAGGAC
TATAAAGATACCAGGCGTTTCCCCCTGGAAGCTCCCTCGTGCGCTCTCCTGTTCCGACCCTGCC
GCTTACCGGATACCTGTCCGCCTTTCTCCCTTCGGGAAGCGTGGCGCTTTCTCATAGCTCACGC
TGTAGGTATCTCAGTTCGGTGTAGGTCGTTCGCTCCAAGCTGGGCTGTGTGCACGAACCCCCCG
TTCAGCCCGACCGCTGCGCCTTATCCGGTAACTATCGTCTTGAGTCCAACCCGGTAAGACACGA
CTTATCGCCACTGGCAGCAGCCACTGGTAACAGGATTAGCAGAGCGAGGTATGTAGGCGGTGCT
ACAGAGTTCTTGAAGTGGTGGCCTAACTACGGCTACACTAGAAGAACAGTATTTGGTATCTGCG
CTCTGCTGAAGCCAGTTACCTTCGGAAGAAGAGTTGGTAGCTCTTGATCCGGCAAACAAACCAC
CGCTGGTAGCGGTGGTTTTTTTGTTTGCAAGCAGCAGATTACGCGCAGAAAAAAAGGATCTCAA

GAAGATCCTTTGATCTTTTCTACGGGGTCTGACGCTCAGTGGAACGAAAACTCACGTTAAGGGA
TTTTGGTCATGAGATTATCAAAAAGGATCTTCACCTAGATCCTTTTAAATTAAAAATGAAGTTT
TAAATCAATCTAAAGTATATATGTGTAACATTGGTCTAGTGATTAGAAAAACTCATCGAGCATC
AAATGAAACTGCAATTTATTCATATCAGGATTATCAATACCATATTTTTGAAAAAGCCGTTTCT
GTAATGAAGGAGAAAACTCACCGAGGCAGTTCCATAGGATGGCAAGATCCTGGTATCGGTCTGC
GATTCCGACTCGTCCAACATCAATACAACCTATTAATTTCCCCTCGTCAAAAATAAGGTTATCA
AGTGAGAAATCACCATGAGTGACGACTGAATCCGGTGAGAATGGCAAAAGTTTATGCATTTCTT
TCCAGACTTGTTCAACAGGCCAGCCATTACGCTCGTCATCAAAATCACTCGCATCAACCAAACC
GTTATTCATTCGTGATTGCGCCTGAGCGAGACGAAATACGCGATCGCTGTTAAAAGGACAATTA
CAAACAGGAATCGAATGCAACCGGCGCAGGAACACTGCCAGCGCATCAACAATATTTTCACCTG
AATCAGGATATTCTTCTAATACCTGGAATGCTGTTTTCCCTGGGATCGCAGTGGTGAGTAACCA
TGCATCATCAGGAGTACGGATAAAATGCTTGATGGTCGGAAGAGGCATAAATTCCGTCAGCCAG
TTTAGTCTGACCATCTCATCTGTAACAACATTGGCAACGCTACCTTTGCCATGTTTCAGAAACA
ACTCTGGCGCATCGGGCTTCCCATACAATCGGTAGATTGTCGCACCTGATTGCCCGACATTATC
GCGAGCCCATTTATACCCATATAAATCAGCATCCATGTTGGAATTTAATCGCGGCCTTGAGCAA
GACGTTTCCCGTTGAATATGGCTCATAACACCCCTTGTATTACTGTTTATGTAAGCAGACAGTT
TTATTGTTCATGATGATATATTTTTATCTTGTGCAATGTAACATCAGAGATTTTGAGACACAAC
GTGGCTTTGTTGAATAAATCGAACTTTTGCTGAGTTGAAGGATCAGATCACGCATCTTCCCGAC
AACGCAGACCGTTCCGTGGCAAAGCAAAAGTTCAAAATCACCAACTGGTCCACCTACAACAAAG
CTCTCATCAACCGTGGCTCCCTCACTTTCTGGCTGGATGATGGGGCGATTCAGGCGATCCCCAT
CCAACAGCCCGCCGTCGAGCGGGCT 10137

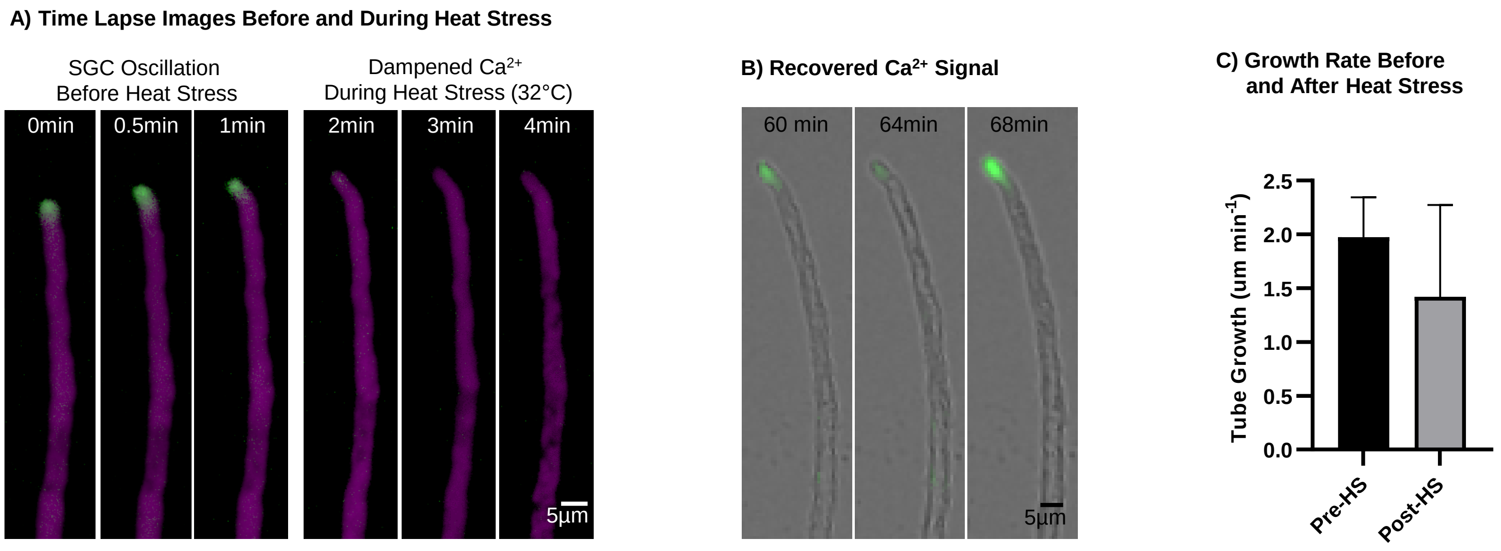

Supplemental Figure S6. Heat stress triggered dampened calcium oscillations do not indicate pollen cell death. (A) Representative time lapse images showing steady growth tip-focused Ca^{2+} (SGC) oscillations shifting to a dampened Ca^{2+} (DC) oscillation during heat stress. Time course: 22°C for 1min, 22-32°C in 1min, followed by 2min at 32°C. **(B)** Tip-focused Ca^{2+} oscillations (green) are restored within 1hour post-heat stress, as shown by representative GFP/brightfield overlay images of the same pollen tube shown in pane A. **(C)** Post-heat stress (post-HS) growth rates are similar to growth rates observed prior to heat stress (pre-HS). Heat treated pollen tubes recovered in the dark at 22°C for ~1hour prior to measuring pollen tube growth over a 10min time course. Error bars are standard deviation of n=6 pollen tubes.
